# Towards Surface‐Enhanced Homogeneous Catalysis: Tailoring the Enrichment of Metal Complexes at Ionic Liquid Surfaces

**DOI:** 10.1002/anie.202422693

**Published:** 2025-02-19

**Authors:** Daniel Hemmeter, Marco Haumann, Federico J. Williams, Thomas M. Koller, Peter Wasserscheid, Karsten Meyer, Florian Maier, Hans‐Peter Steinrück

**Affiliations:** ^1^ Lehrstuhl für Physikalische Chemie 2 Friedrich-Alexander-Universität Erlangen-Nürnberg Egerlandstr. 3 91058 Erlangen Deutschland; ^2^ Friedrich-Alexander-Universität Erlangen-Nürnberg (FAU) Lehrstuhl für Chemische Reaktionstechnik (CRT), Egerlandstr. 3 91058 Erlangen Deutschland; ^3^ Research Centre for Synthesis and Catalysis Department of Chemistry University of Johannesburg P.O. Box 524, Auckland Park 2006 South Africa; ^4^ Departamento de Química Inorgánica Analítica y Química Física Facultad de Ciencias Exactas y Naturales Universidad de Buenos Aires Buenos Aires Argentina; ^5^ Instituto de Química Física de los Materiales Medio Ambiente y Energía CONICET-Universidad de Buenos Aires Buenos Aires Argentinien; ^6^ Friedrich-Alexander-Universität Erlangen-Nürnberg (FAU), Lehrstuhl für Advanced Optical Technologies – Thermophysical Properties (AOT-TP) Paul-Gordan-Straße 8 91052 Erlangen Deutschland; ^7^ Forschungszentrum Jülich GmbH Helmholtz-Institute Erlangen-Nürnberg (IET-2) Cauerstr. 1 91058 Erlangen; ^8^ Institute for a Sustainable Hydrogen Economy Marie-Curie-Straße 5 52428 Jülich Deutschland; ^9^ Friedrich-Alexander-Universität Erlangen-Nürnberg (FAU) Department für Chemie und Pharmazie, Anorganische Chemie Egerlandstraße 1 91058 Erlangen Deutschland

**Keywords:** homogeneous catalysis, ionic liquids, surface enrichment, supported ionic liquid phase (SILP) catalysis, angle-resolved X-ray photoelectron spectroscopy (ARXPS)

## Abstract

When talking about homogeneous catalyst systems, it has long been assumed that the system at hand consists of a transition metal complex in solution with the liquid interface representing the composition of the bulk solution. Now, in light of considerable developments in the study of metal complexes dissolved in ionic liquids with their negligible vapor pressures, more detailed studies of the composition at the liquid/gas interface became possible. These investigations revealed pronounced surface enrichment and segregation effects of high relevance for practical applications. This article reviews recent advancements in tailoring the interfacial composition of ionic liquid‐based catalytic systems. A particular focus is dedicated to surface enrichment phenomena, and a variety of parameters are presented for deliberate control of the local concentration of the complexes at the surface, that is, the nature of the ligands, the bulk concentration, the temperature, and the nature of the IL solvent. As experimental methods, angle‐resolved X‐ray photoelectron spectroscopy (ARXPS) and vacuum‐based pendant‐drop surface tension measurements were applied. The reviewed results are intended to provide the basis for the advancement of catalytic systems with high surface areas, such as in supported ionic liquid phase (SILP) catalysis, where the interface design is directly interconnected with catalytic performance.

## Graphical abstract

The article reviews strategies to deliberately anchor metal complexes at the liquid/vacuum (gas) interface of ionic liquids in a buoy‐like fashion; this surface enrichment is achieved by targeted functionalization of the catalytic complexes with surface‐active ligands and the appropriate choice of the ionic liquid.

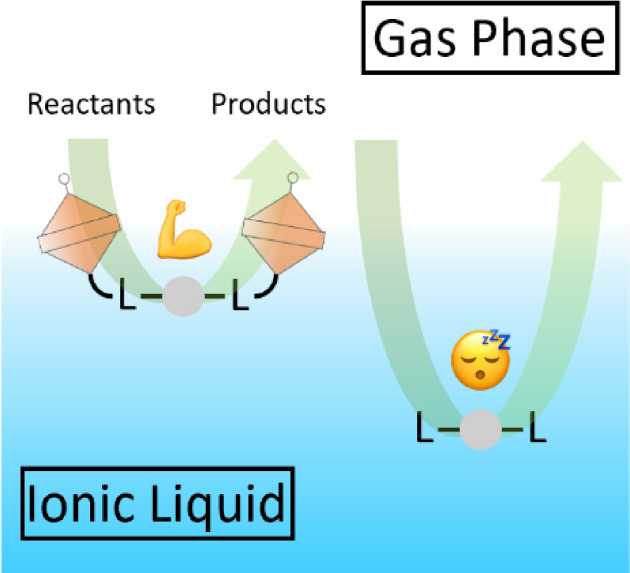

## Introduction

The need for sustainable manufacturing and processing of materials across the value chain substantially shapes modern science and technology. One major objective to be addressed is the comprehensive transformation of the chemical industry towards processes with efficient avoidance or recycling of waste and secondary products.^[1]^ With a special focus on atom‐economical and selective conversions, it is safe to say that the development, optimization, and upscaling of novel catalytic systems play a decisive role in this paradigm change.^[2]^

The overall performance of catalysts is essentially influenced by their microscopic nature, that is, composition, structure, and electronic properties of the active sites.[^3^, ^4^] Technical heterogeneous catalysts commonly feature a non‐uniform and defect‐rich surface structure and thus an intricate spectrum of catalytic centers differing in activity and selectivity.^[5]^ Especially for the production of more value‐added products, the potentially low selectivity can pose economic and ecological limitations, owing to the required separation of the product mixture.^[6]^ Despite significant progress in increasing the selectivity of heterogeneous catalysts in recent decades,[^7^, ^8^, ^9^] homogeneous organometallic catalysis has gained increasing appeal providing uniform reactivity with well‐accessible information on structure‐performance relationships.^[10]^ Based on this knowledge, the structural variability of organometallic chemistry in solution enables the deliberate design of the catalysts and, thus, sensitive control over activity and selectivity on the molecular level. However, in contrast to heterogeneous systems, costly procedures for catalyst separation and recovery can limit lucrative applications.^[11]^

**Figure 1:**
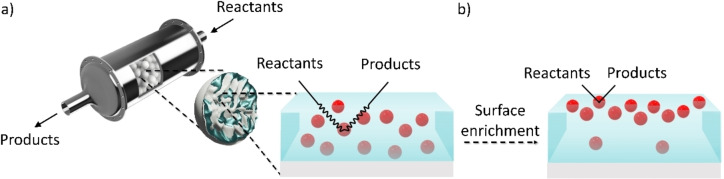
Schematic representation of the supported liquid phase (SLP) concept (for ionic liquids: supported ionic liquid phase, SILP a) from the macroscopic (left) to the microscopic scale (right) and b) reduction of the diffusion pathways of reactants and products upon enrichment of the catalytically active species (red spheres) at the liquid/gas interface.

Intending to combine the facile engineering of heterogeneous systems and the powerful catalytic performance of metal complexes, techniques for immobilization of homogeneous catalysts in product‐separable phases have received significant attention.[^12^, ^13^] One intensively investigated approach is the heterogenization of organometallics by physicochemical grafting onto the surface of solid support materials, e.g. by covalent bonding^[14]^ or electrostatic interactions.^[15]^ However, the static fixation can considerably compromise the tunability and characterizability and can negatively affect activity and selectivity[^16^, ^17^] – the principal advantages of homogeneous organometallic catalysis. Consequently, the productivity of these systems for commercial use has been questioned.^[17]^ An alternative strategy addresses the immobilization of the homogeneous catalyst in a supported liquid phase (SLP).[^18^, ^19^] For this, a solid, high‐surface area support is impregnated with a thin film of catalyst solution. The macroscopic properties of the catalytic system are still governed by the powdery support material, allowing for the favorable reaction engineering of heterogeneous catalysis with efficient separation of catalyst and products. Within the immobilized liquid film, however, the well‐defined homogeneous surrounding principally grants the full spectrum of chemical preferences provided by organometallic catalysis. This concept is illustrated in Figure 1a.

The materials class of ionic liquids (ILs) offers immense scope for scientific and technological innovation for this type of catalyst immobilization, termed the supported ionic liquid phase (SILP) concept.[^20^, ^21^] ILs are liquid salts, typically comprising bulky organic cations and/or anions with low charge density, yielding low melting points, oftentimes below room temperature. Given their ionic character, ILs show extremely low vapor pressures and, therefore, intrinsically provide coatings with excellent persistence toward leaching into the surrounding gas phase, even after extensive time‐on‐stream, e.g. more than 800 h in Rh‐catalyzed hydroformylation.^[22]^ The mostly organic backbone of the ions, on the other hand, enables task‐specific molecular design for deliberate tuning of physicochemical properties for optimum performance regarding, for instance, solvation and coordination behavior, hydrophobicity, miscibility, and wetting capability. SILP systems have been successfully trialed for various other industry‐relevant conversions, such as hydrogenation,[^23^, ^24^] hydroformylation,[^25^, ^26^] carbonylation,^[27]^ and hydrosilylation,[^28^, ^29^] to name but a few. Beyond that, a study by Werner et al. demonstrated the adaptable character of the SILP concept by systematically varying the IL, the support material, and the catalyst complex, thereby achieving remarkably mild conditions in water‐gas‐shift catalysis.^[30]^

In this context, a fundamental understanding on the nature of the IL/gas interface offers interesting parameters for optimization in SILP catalysis.^[31]^ In principle, a particularly high catalyst concentration directly at the IL/gas interface, where the feedstock concentration is at its maximum, would result in the most efficient catalyst utilization, as is signified in Figure 1b. This benefit becomes particularly obvious considering the high viscosities of ILs and, thus, the much slower diffusion rates of dissolved solutes in solution compared to conventional solvents.[^32^, ^33^, ^34^] Consequently, surface enrichment of the catalyst would minimize the diffusion pathways in the liquid phase. Early works on the composition of the IL/vacuum interface of dissolved metal compounds in ILs in our group have revealed strong enrichment of [Pt(NH_3_)_4_]^2+^ in the IL [C_2_C_1_Im][C_2_OSO_3_], while Cl^−^ counterions were found depleted from the surface using angle‐resolved X‐ray photoelectron spectroscopy (ARXPS).^[35]^ This surface activity was attributed to the higher polarizability of the larger metal‐containing cation.^[35]^ A subsequent study employed the phosphine ligand trinatrium‐3,3′,3′′‐phosphintriyltribenzolsulfonat (TPPTS) as a suitable surface‐active ligand to trigger surface enrichment of a Rh complex, while the precursor complex without TPPTS did not exhibit surface affinity.^[36]^ In a more recent study, the chemical and interfacial behavior of another Rh complex, [Rh(COD)_2_][TfO], in ILs was investigated, and TPPTS was also used to enhance the surface concentration of an *in situ* formed Schrock‐Osborn‐type complex.^[37]^

This article provides an overview of recent results addressing the behavior of organometallic complexes in IL solution at the IL/vacuum interface, with a special focus on enrichment and depletion effects probed by ARXPS. This technique provides detailed insights into the surface composition of the samples and chemical information on the metal complexes under investigation. The latter aspect has already been realized in the pioneering XPS work for a Pd complex dissolved ILs by the group of *Peter Licence* in 2005.^[38]^ Since then, XPS has been intensively used to explore the electronic and interfacial nature of ILs and their mixtures and solutions.[^39^, ^40^, ^41^, ^42^, ^43^, ^44^, ^45^, ^46^, ^47^, ^48^, ^49^, ^50^, ^51^, ^52^] Over the past years, our groups have provided a significant set of data on the surface enrichment of organometallic complexes in ILs, which are highlighted and contextualized together with findings of other groups with different experimental techniques. In particular, we will cover the interfacial behavior and chemical properties of complexes with different ligand systems in IL solution systems, as well as strategies how to deliberately modify the ligands to achieve enrichment at the IL/vacuum interface. Furthermore, the influence of the bulk concentration, the temperature, and the nature of the IL on the local concentration of the complexes at the interface will be discussed. For selected examples, we will cross‐correlate the data and conclusions on the molecular level derived from ARXPS with macroscopic surface tension measurements for various bulk concentrations and analyze our data using the Gibbs adsorption isotherm.

Since the purity of the used ILs is absolutely essential, as are the developed methods and routines for preparing the IL solutions with dissolved metal complexes, we start by addressing these two aspects in the following. After a short experimental account, we then discuss surface orientation and enrichment effects in ILs and their mixtures, which sets the stage for addressing the main topic of this review, that is, the surface enrichment of catalysts with different ligands in IL solutions. Particular focus will be given on the influence of the catalyst bulk concentration, the temperature, and the nature of the solvent IL. The review will close with a conclusion, where we deduce detailed strategies for achieving surface enrichment of the catalyst complexes in general.

The specific systems included in this review have been selected based on the following criteria: 1) The purity of the IL has been verified, the surfaces were free from contaminations (a common challenge, particularly when working with ILs from commercial suppliers), and the surface composition has been characterized. 2) The metal complexes are soluble over a wide composition range; solubility is a challenge for many complex/IL combinations, but can e.g. achieved by introducing side groups/ligands with a similar chemical structure as the supporting IL. 3) The complexes show surface enrichment. 4) Surface enrichment was studied systematically over a wide composition range. 5) We wanted to include charged and neutral complexes, and 6) results on the catalytic performance where available.

## Materials

ILs and metal complexes discussed in this review are presented in Tables 1 and 2, respectively, including abbreviations used, full names (of the ILs), molecular weights, molecular structures, and color‐coded assignment of specific carbon species distinguished in the presented XP spectra. Note that the metal complexes discussed in the following will be referred to as “catalysts”, even though some of the employed complexes might not have proven yet to be catalytically active for a specific reaction.

**Table 1:** Nomenclature, molecular weights and structures of ILs used. The color coding indicates the assignment of carbon species to XP signals (green: C_C2_ or C_C2/COO_ violet: C_hetero_, blue: C_alkyl_).

Abbreviation	Name	Molecular weight /g/mol	Molecular Structure
[C_2_C_1_Im][PF_6_]	1‐Ethyl‐3‐methylimidazolium hexafluorophosphate	256.13	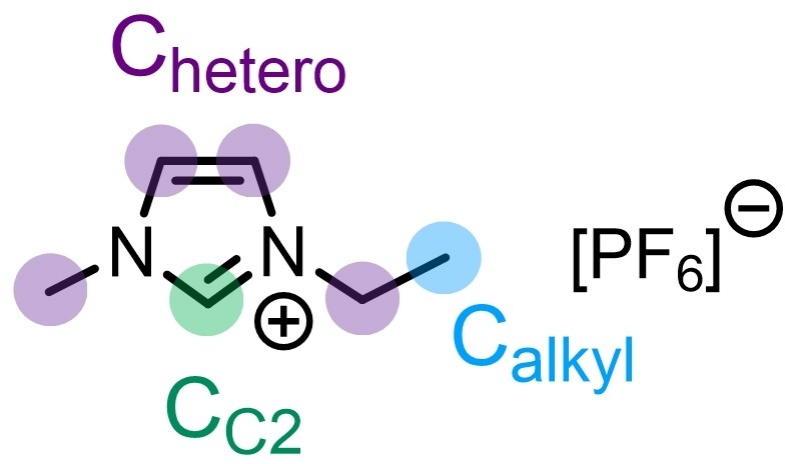
			
[C_2_C_1_Im][OAc]	1‐Ethyl‐3‐methylimidazolium acetate	170.21	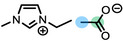
			
[C_2_C_1_Im][TfO]	1‐Ethyl‐3‐methylimidazolium trifluormethansulfonate	260.24	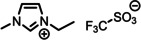
			
[C_2_C_1_Im][C_2_OSO_3_]	1‐Ethyl‐3‐methylimidazolium ethylsulfate	236.29	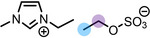
			
[C_4_C_1_Im][PF_6_]	1‐Butyl‐3‐methylimidazolium hexafluorophosphate	284.18	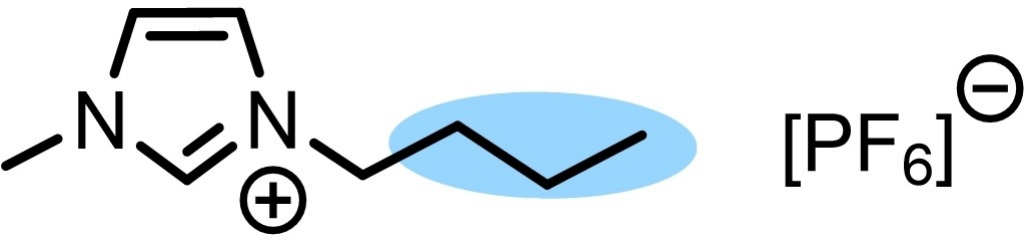
			
[C_4_C_1_Im][Tf_2_N]	1‐Butyl‐3‐methylimidazolium bis(trifluormethylsulfonyl)imide	419.37	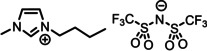
			
[C_4_C_1_Im][TfO]	1‐Butyl‐3‐methylimidazolium trifluormethansulfonate	288.29	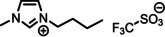
			
[C_4_C_1_Im]Cl	1‐Butyl‐3‐methylimidazolium chloride	174.68	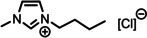
			
[C_8_C_1_Im][PF_6_]	1‐Methyl‐3 octylimidazolium hexafluorophosphate	340.29	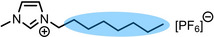
			
[C_8_C_1_Im][TfO]	1‐Methyl‐3‐octylimidazolium trifluormethansulfonate	344.40	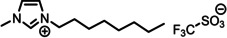
			
[C_3_CNC_1_Im][Tf_2_N]	1‐(3‐Cyanopropyl)‐3‐ methylimidazolium bis‐ (trifluormethylsulfonyl)imide	430.34	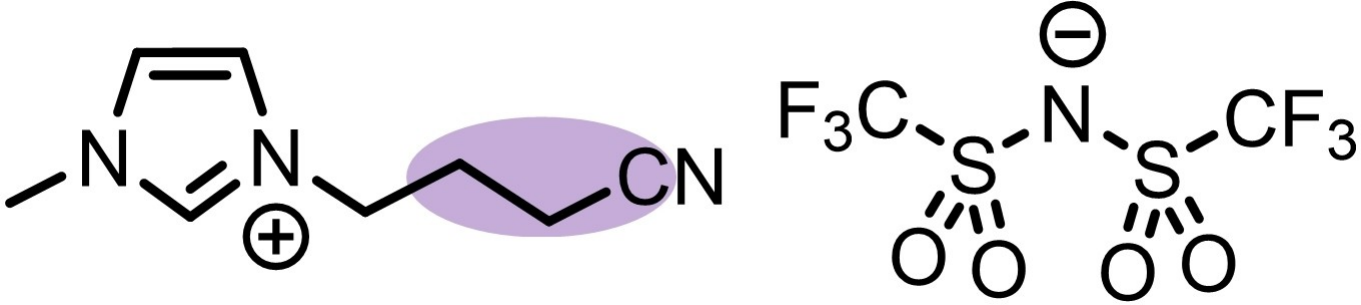
			
[C_1_CNC_1_Pip][Tf_2_N]	1‐(1‐Cyanomethyl)‐1‐ methylpiperidinium bis‐ (trifluormethylsulfonyl)imide	419.36	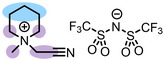
			
[C_3_CNC_1_Im][PF_6_]	1‐(3‐Cyanopropyl)‐3‐ methylimidazolium hexafluorophosphate	295.16	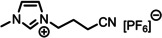
			
[C_3_CNPFC_4_Im][PF_6_]	1‐(3‐Cyanopropyl)‐3‐ (3,3,4,4,4‐pentafluorbutyl) imidazolium hexafluorophosphate	427.20	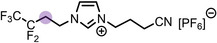
			
[(mPEG_2_)_2_Im] I	1,3‐Bis(2‐(2‐methoxy‐ethoxy)ethyl)‐ imidazolium iodide	400.26	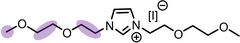
			
[(mPEG_2_)_2_Im][PF_6_]	1,3‐Bis(2‐(2‐methoxy‐ethoxy)ethyl)‐ imidazolium hexafluorophosphate	418.31	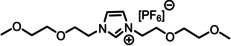
			

**Table 2:** Molecular structures and weights of complexes used. The color coding indicates the assignment of carbon species to XPS signals (green: C_C2_ or C_C2/COO_ violet: C_hetero_, blue: C_alkyl_). For assigment of IL‐derived ligands (complexes **1**–**5**) and structure of the [Tf_2_N]^−^ anion, see Table 1.

Formula	Abbreviation used in this thesis	Molecular weight /g/mol	Molecular Structure
[PtCl_2_(C_3_CNC_1_Im)_2_][Tf_2_N]_2_	**1**	1126.66	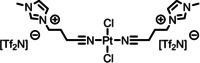
			
[PdCl_2_(C_3_CNC_1_Im)_2_][Tf_2_N]_2_	**2**	1038.00	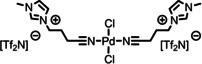
			
[PtCl_2_(C_1_CNC_1_Pip)_2_][Tf_2_N]_2_	**3**	1104.70	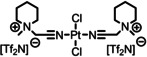
			
[PtCl_2_(C_3_CNC_1_Im)_2_][PF_6_]_2_	**4**	856.30	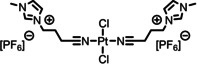
			
[PtCl_2_(C_3_CNPFC_4_Im)_2_][PF_6_]_2_	**5**	1120.38	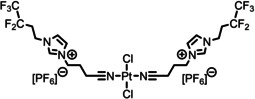
			
[Ru(tpy)(bpy)Cl][PF_6_]	**6**	670.94	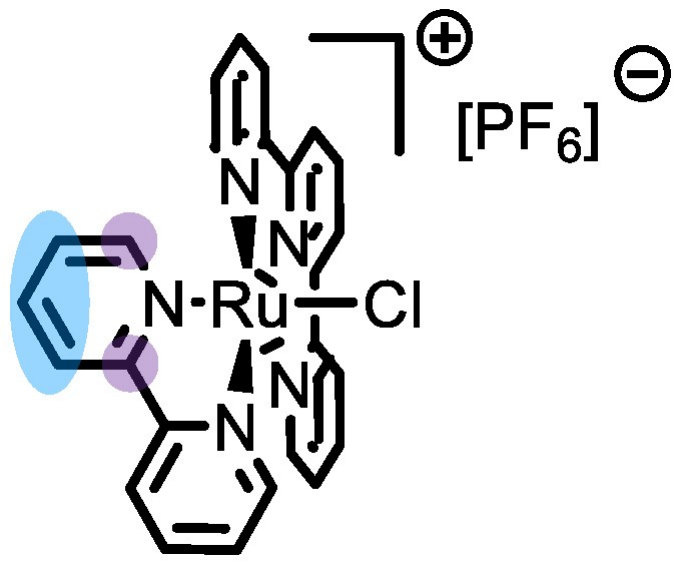
			
[Ru(tpy)(dcb)Cl][PF_6_]	**7**	758.96	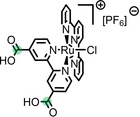
			
[Ru(dcbNa)_2_((C_9_)_2_bpy)][PF_6_]_2_	**8**	1376.01	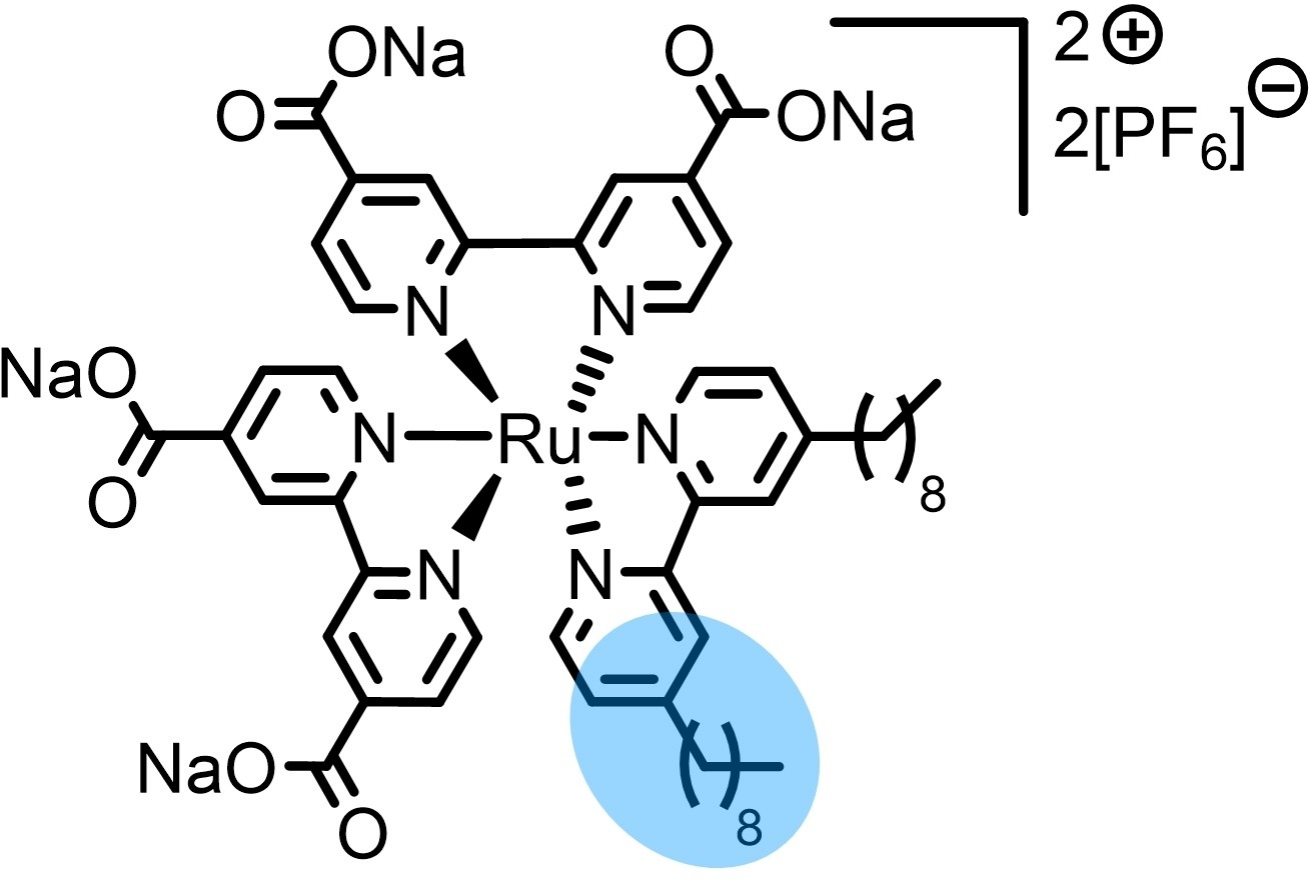
			
[Ru(dcbNa)_2_((C_1_)_2_bpy)][PF_6_]_2_	**9**	1151.58	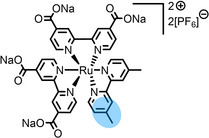
			
[Ru(dcbNa)_2_((OC_2_)_2_bpy)][PF_6_]_2_	**10**	1211.63	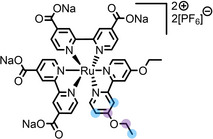
			
[Ru(dcbNa)_2_((t‐C_4_)_2_bpy)][PF_6_]_2_	**11**	1235.74	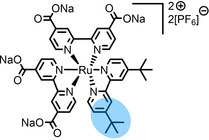
			
trans‐[PtCl_2_(mPEG_3_C_8_Im)_2_]	**12**	918.93	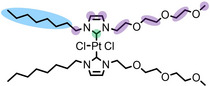
			
[Rh(COD)_2_][TfO]	**13**	468.34	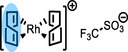
			
[Rh(COD)(TPPTS)_2_][TfO]	**14**	1497.00	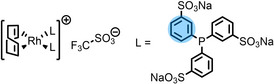
			

Some of the ILs addressed in this review showed surface‐active contaminations, which were clearly discernible using ARXPS, e.g. by unexpectedly high C_alkyl_ and O 1s signals (at 0° and even more at 80°; for experimental details of ARXPS, see below).[^53^, ^54^] As contaminations can largely limit the meaningfulness of surface‐sensitive experiments,^[55]^ the respective ILs were cleaned by various procedures.[^53^, ^54^] All other ILs were used as received. The absence of common Si‐based surface‐active contaminations^[55]^ was checked for every studied IL or solution using ARXPS.

**Scheme 1:**
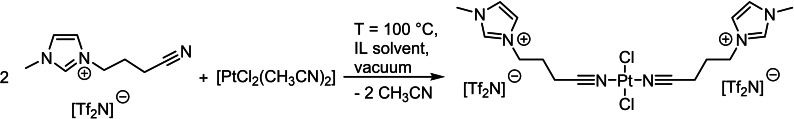
Preparation of complex **1** in excess of [C_3_CNC_1_Im][Tf_2_N], which acts as both solvent and ligand. Complexes **1**–**5** were prepared in a similar way also directly in other ILs.[^53^, ^56^, ^57^, ^60^] Note that also non‐functionalized ILs were successfully applied as solvents, so that the CN‐functionalized ligand IL was reacted in stoichiometric amounts with the metal precursor.[^53^, ^57^, ^60^]

The detailed synthetic routes of the studied functionalized ILs can be found in Refs.[^53^, ^56^, ^57^, ^58^, ^59^] In the following, we briefly summarize some of the procedures applied to achieve well‐defined clean surfaces. Solutions, where a solid catalyst or ligand was available, were typically prepared by stirring the solute in the IL for several hours under ambient conditions. For preparation of solutions of complexes **1**–**5**, the following general procedure applies (for additional details see Refs.^[53,56,57,60^): Mixtures of the precursor cis‐[PtCl_2_(CH_3_CN)_2_] or [PdCl_2_(CH_3_CN)_2_] and the ligand were reacted in stoichiometric amounts in the respective IL at 100 °C under medium vacuum (MV) conditions using Schlenk‐techniques until the metal precursor was fully consumed and the released volatile CH_3_CN ligands were fully removed yielding clear solutions (see also Scheme 1). Typically, the solutions were further stirred for 1 h under the applied conditions to ensure quantitative conversion. For solutions of **5**, *cis*‐[PtCl_2_(CH_3_CN)_2_] and the ligand IL [C_3_CNPFC_4_Im][PF_6_] were first reacted without additional IL solvent for 1 h under the conditions applied above until solidification of the mixture, before adding the IL solvent to continue the reaction as described above.[^53^, ^57^, ^60^] 1 %_mol_ solutions were prepared by simply diluting freshly prepared, more concentrated solutions.[^53^, ^60^] For a 1 %_mol_ solution of **5** in [C_4_C_1_Im][PF_6_] and solutions of **5** in [C_2_C_1_Im][PF_6_], strong X‐ray‐induced decomposition of the complex was observed due to the presence of traces of iodine species in the ILs.[^53^, ^60^] To prevent this effect, the ILs were cleaned by extraction with Millipore water (resistivity 18.2 MΩ ⋅ cm), prior to the preparation of the catalyst solutions.^[53]^ Pre‐emptively, the cleaning procedure was also applied to all other ILs to prepare the 1 %_mol_ solutions of **5** and a 10 %_mol_ of **5** in [C_4_C_1_Im][Tf_2_N].^[60]^ For successful preparation of a 33.3 %_mol_ solution of **3** in [C_1_CNC_1_Pip][Tf_2_N], UHV conditions were required, while in MV, solidification of the entire mixture upon reaction progress was observed.^[56]^ In the case of the ligand substitution reaction of **12** with TPPTS in [C_2_C_1_Im][C_2_OSO_3_], TPPTS was dissolved under inert gas conditions for 70 h before **12** was added, and the reaction mixture was stirred under vacuum for 24 h to yield a clear solution.^[37]^

## Experimental Aspects

The data provided in this review are mostly determined by X‐ray photoelectron spectroscopy (XPS) using monochromated^[61]^ (and for one system non‐monochromated)^[62]^ Al−Kα X‐radiation (hν=1486.6 eV). XPS yields quantitative insights into the composition of the near‐surface region along with chemical information, e.g. on oxidation state, bonding conditions, and other interactions of the atoms under investigation. The surface sensitivity of XPS can be tuned by varying the electron emission angle ϑ – a technique referred to as angle‐resolved XPS (ARXPS). At 0° (normal emission), the ID is 6–9 nm in organic materials,^[61]^ corresponding to several molecular layers in the surface‐near region of the sample. At 80° (grazing emission), the ID decreases to 1.0–1.5 nm, which mainly reflects the topmost molecular layer of the sample.^[61]^

The investigated solutions were applied, unless stated otherwise, under ambient conditions onto the setup‐compatible sample holders^[61]^ as a ~0.5 mm thick film; for these macroscopically thick films, the IL/solid interface did not affect the composition at the IL/vacuum interface. Owing to this fact (except where stated otherwise) “surface” corresponds to the IL/vacuum interface throughout this review. In the case of solid residuals, the particles settled to the ground of the sample holder and were expected not to affect the measurements. Solid IL samples were typically applied as hot liquids (~80 °C) after melting and thoroughly degassing under MV conditions using standard Schlenk techniques.

**Figure 2:**
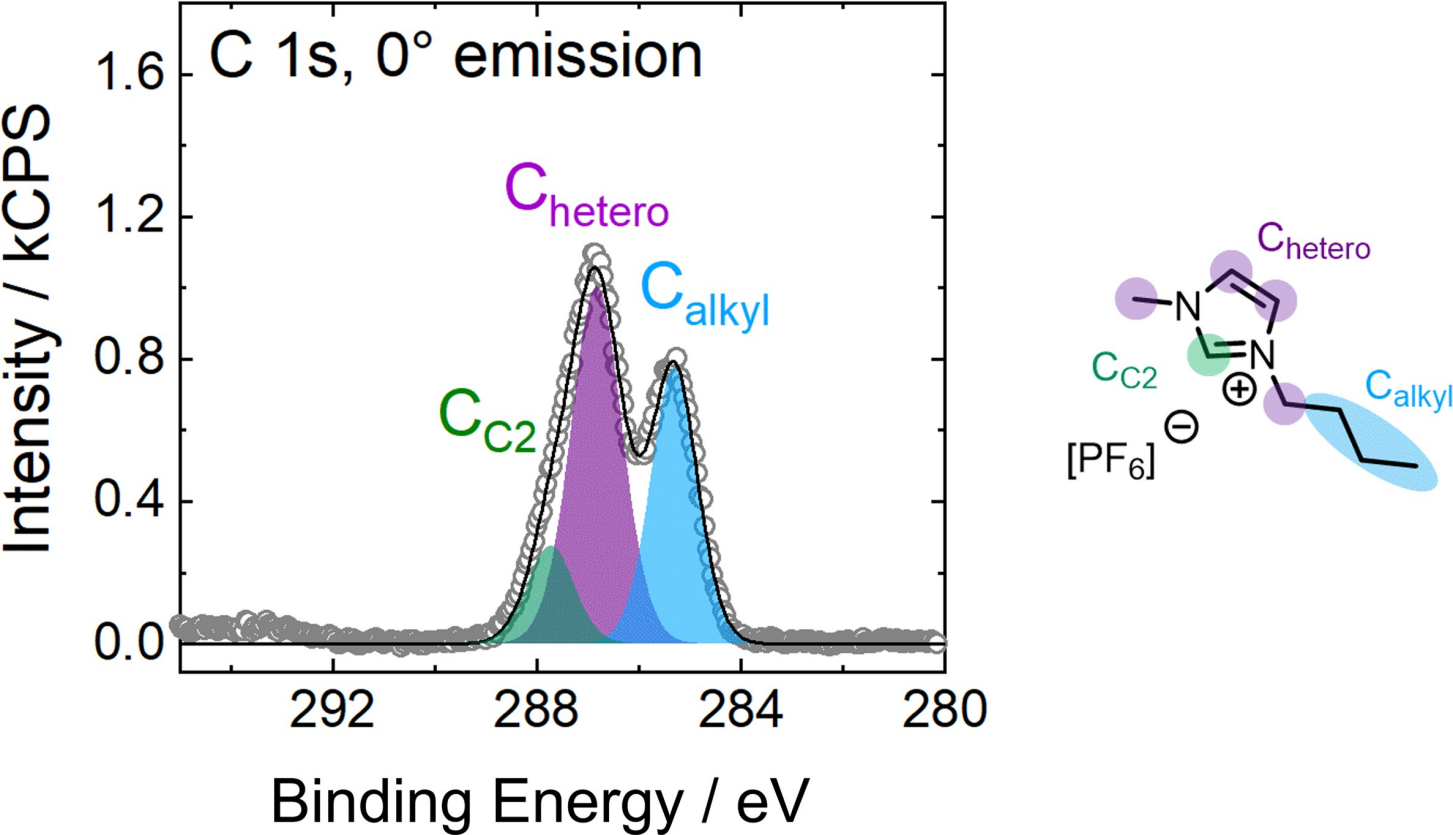
C 1s spectrum of neat [C_4_C_1_Im][PF_6_] in 0° emission with color‐coded deconvolution and assignment to the molecular structure.

Detailed procedures for fitting and referencing of the recorded ARXPS data can be found in Refs.,[^37^, ^53^, ^56^, ^57^, ^60^, ^63^, ^64^, ^65^] following an established procedure for neat ILs investigated with the DASSA setup,^[61]^ which was expanded also to the solutions presented herein. The typically applied deconvolution procedure for the broad C 1s signal envelope from ~289 to 284 eV is exemplified in Figure 2, with the assignment of C atoms to XPS signals shown color‐coded in Tables 1 and 2. The deconvolution procedures involved distinction of three different types of C atoms contributing to the signal envelope: C atoms bound to two heteroatoms within the imidazolium ring, C_C2_ (green), C atoms bound to one heteroatom, C_hetero_ (violet), and C atoms bound to only other carbon atoms and hydrogen, C_alkyl_ (blue). The derived intensities are expected to reflect the actual surface composition within an uncertainty range of 5–10 %. Raw peak intensities were corrected using atomic sensitivity factors (ASFs) derived from Ref.^[66]^ To compensate for the inherently lower overall intensity detected at grazing emission, the 80° spectra were multiplied by a geometry correction factor.^[61]^ This procedure facilitates the comparison of individual peaks for deducing orientational and enrichment effects.

The surface tension values were assessed using the pendant drop method. The measurements were performed in a novel chamber under ultraclean vacuum conditions down to the 10^−7^ mbar region, minimizing the influence of moisture or dissolved gases.^[67]^ The cleanliness of the ILs and catalyst solutions was checked before the measurements using XPS.

## Surface Orientation and Enrichment Effects in Ionic Liquids and their Mixtures

As a result of the anisotropic environment at the surface, ions and molecules located in the surface‐near region can show distinct structural and molecular ordering to minimize the surface free energy. This ordering can appear (1) in the form of orientational phenomena at the surface, that is, preferred orientations, configurations, and alignment of ions, molecules, or certain functional groups, or (2) in the form of surface enrichment phenomena in IL mixtures or solutions, which are a deviation of the local concentration of ions and molecules at the surface relative to the average bulk concentration. Since orientational and enrichment phenomena can appear simultaneously and are superimposed in the XP spectra of our solutions presented herein, we will illustrate these effects along instructive examples of the neat IL [C_8_C_1_Im][ClC_4_SO_3_]^[62]^ and binary mixtures of [C_4_C_1_Im][PF_6_] and the fluorinated analog [PFC_4_C_1_Im][PF_6_]^[68]^ in the following.

**Figure 3:**
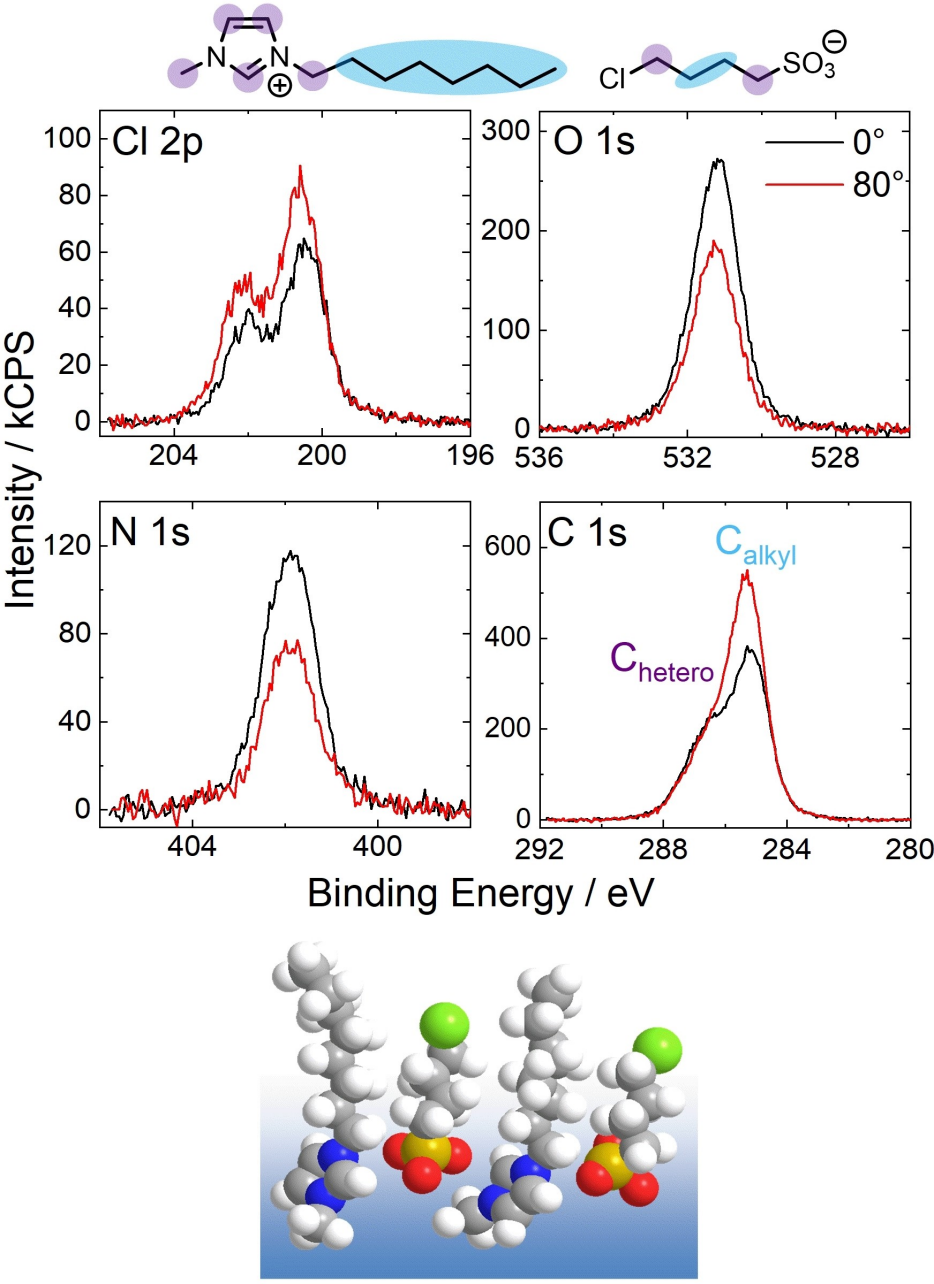
Cl 2p, N 1s, O 1s, and C 1s XP spectra of neat [C_8_C_1_Im][ClC_4_SO_3_] in 0° (black) and 80° emission (red) at room temperature and molecular structure with color‐coded assignment of carbon species deconvoluted in XPS. Note that the employed instrument differs^[62]^ from the instrument used to acquire all other spectra presented in this review. Additionally, the derived surface orientation of the ions is schematically presented. Reproduced from Ref.^[43]^ with permission from the PCCP Owner Societies.

Figure 3 shows relevant XP spectra recorded from neat [C_8_C_1_Im][ClC_4_SO_3_] at 0° (black, more bulk‐sensitive) and 80° emission (red, more surface‐sensitive). Comparing the spectra in these two geometries demonstrates the preferred surface orientations of both ions: While the spin‐orbit‐resolved Cl 2p signals increase at 80°, the O 1s signal strongly decreases, unambiguously revealing that the Cl tail of the [ClC_4_SO_3_]^−^ anion is directed towards the vacuum, while the SO_3_^−^ groups point toward the bulk. A similar orientational effect can be easily observed for the [C_8_C_1_Im]^+^ cation, when considering the N 1s and C 1s spectra. The N 1s signal showed a decrease, and – by contrast – the C_alkyl_ signal, with its major contribution from the C_8_ chain, shows a strong increase when increasing the surface sensitivity. These findings revealed that the C_8_ chains terminate the surface, while the charged headgroups are located more distant from the surface.^[62]^ The orientation of both ions is schematically presented at the bottom of Figure 3.[^43^, ^62^]

**Figure 4:**
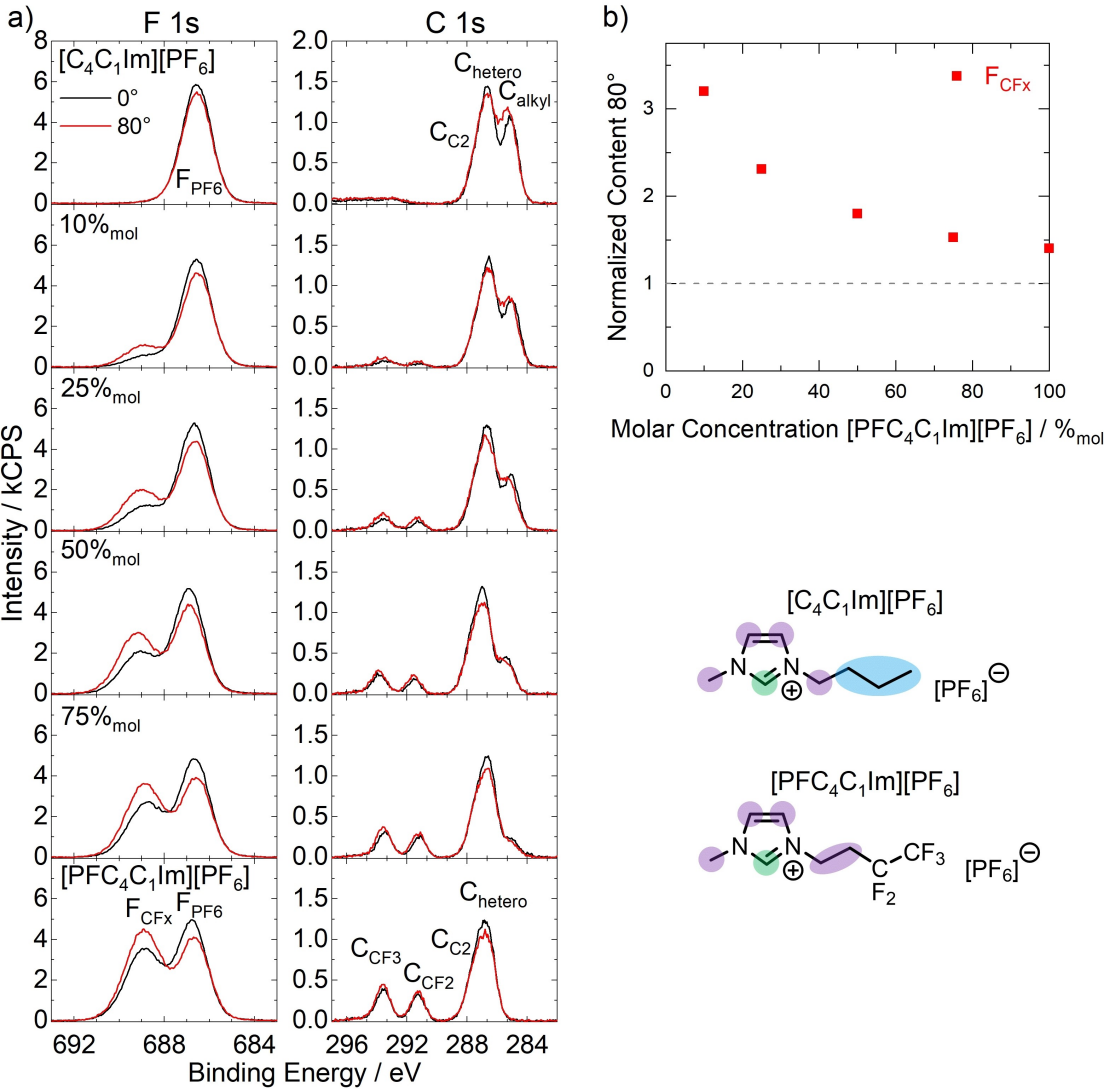
a) F 1s and C 1s XP spectra of binary mixtures of [C_4_C_1_Im][PF_6_] and [PFC_4_C_1_Im][PF_6_] with different [PFC_4_C_1_Im][PF_6_] concentrations and b) normalized content of F_CFx_ (red squares) in 80° emission plotted against the nominal/bulk concentration. The sample temperature was 95 °C in all cases. The grey dashed horizontal line indicates a situation with nominal composition at the surface, that is, in the absence of enrichment effects and preferential orientations of the ions. Adapted from Ref.^[68]^

Preferential orientations are also observed for neat [C_4_C_1_Im][PF_6_] and [PFC_4_C_1_Im][PF_6_] ILs:^[68]^ In the C 1s spectrum of neat [C_4_C_1_Im][PF_6_] (*top C 1s* in Figure 4a), the C_alkyl_ signal of the C_4_ chain shows an enhancement at 80°, which is due to the C_4_ chains being directed toward the vacuum at the surface. This effect is well‐studied and known for ILs with extended alkyl chains.[^42^, ^43^, ^69^, ^70^, ^71^, ^72^, ^73^, ^74^] [PFC_4_C_1_Im][PF_6_] also exhibits a preferred surface orientation, with the PFC_4_ chains pointing toward the vacuum. This effect is best evident from the F 1s spectrum of the neat IL (*bottom F 1s* in Figure 4a), which shows a pronounced increase of the F_CFx_ signal at 80°, while at the same time, the F_PF6_ signal of the anions decreases. For the binary mixtures of the two ILs, a surface enrichment of the fluorinated IL was observed at all concentrations studied. This enrichment was evident from an overall higher F_CFx_ intensity than expected from the nominal composition at 0°, and pronounced signal increase at 80°. At the same time, the C_alkyl_ consistently showed a lower intensity than expected from the nominal composition at all concentrations at 0° and remained constant or showed a slight decrease at 80° due to the enrichment of [PFC_4_C_1_Im][PF_6_] and the associated surface depletion of [C_4_C_1_Im][PF_6_]. The surface enrichment of [PFC_4_C_1_Im][PF_6_] is also evident from Figure 4b, where the normalized F_CFx_ content (red), that is, the experimentally observed intensity at 80° divided by the nominally expected intensity, is shown for all concentrations: The increase of the normalized F_CFx_ content with decreasing concentration indicates a higher degree of enrichment at lower [PFC_4_C_1_Im][PF_6_] concentrations.^[68]^ This pronounced surface affinity of the PFC_4_ chains served as the basis for the deliberate enrichment of a Pt catalyst presented in the following.

## Surface Composition of Catalysts with different Ligands in Ionic Liquid Solution

### Catalysts with Ionic Liquid‐Derived Ligand Systems

A fundamental requirement for studying catalyst solutions using ARXPS is the sufficient solubility of the catalyst in the IL. While for catalytic conversions a relatively low solubility in the ppm range is typically acceptable, adequate intensities can only be detected in ARXPS with a (surface) concentration of, as a rule of thumb, ≥1 %_mol_. Introducing IL building blocks into the ligand system was an intuitive approach to obtain complexes that are highly soluble in various ILs. With respect to potential catalytic applications, such charge‐tagged complexes have shown a particularly high resistance against catalyst leaching, including ‐ among many examples[^75^, ^76^] – catalysts with nitrile‐functionalized IL cations as ligands.[^77^, ^78^, ^79^] The following paragraphs describe the formation of such catalysts, their interfacial behavior, and the rational design of the ligand system for deliberate surface enrichment, as well as a short discussion of the impact of the surface enrichment in hydrogenation of ethene.

Previous preparation routes for Pd complexes with IL‐derived ligands were based on the reaction of PdCl_2_ and stoichiometric amounts of the ligand at room temperature and ambient pressure in volatile solvents such as dichloromethane or acetonitrile, as reported by the *Dyson* group.[^77^, ^78^, ^79^] We now used a very different pathway directly in the respective IL solvent under vacuum conditions:^[56]^ Scheme 1 demonstrates this approach exemplarily for the formation of the Pt complex **1** in [C_3_CNC_1_Im][Tf_2_N] from the metal precursor *cis*‐[PtCl_2_(CH_3_CN)_2_]. The concept of this route is the immediate removal of CH_3_CN ligands from the reaction vessel by the vacuum pump after ligand substitution by the IL cation at elevated temperature (100 °C).

**Figure 5:**
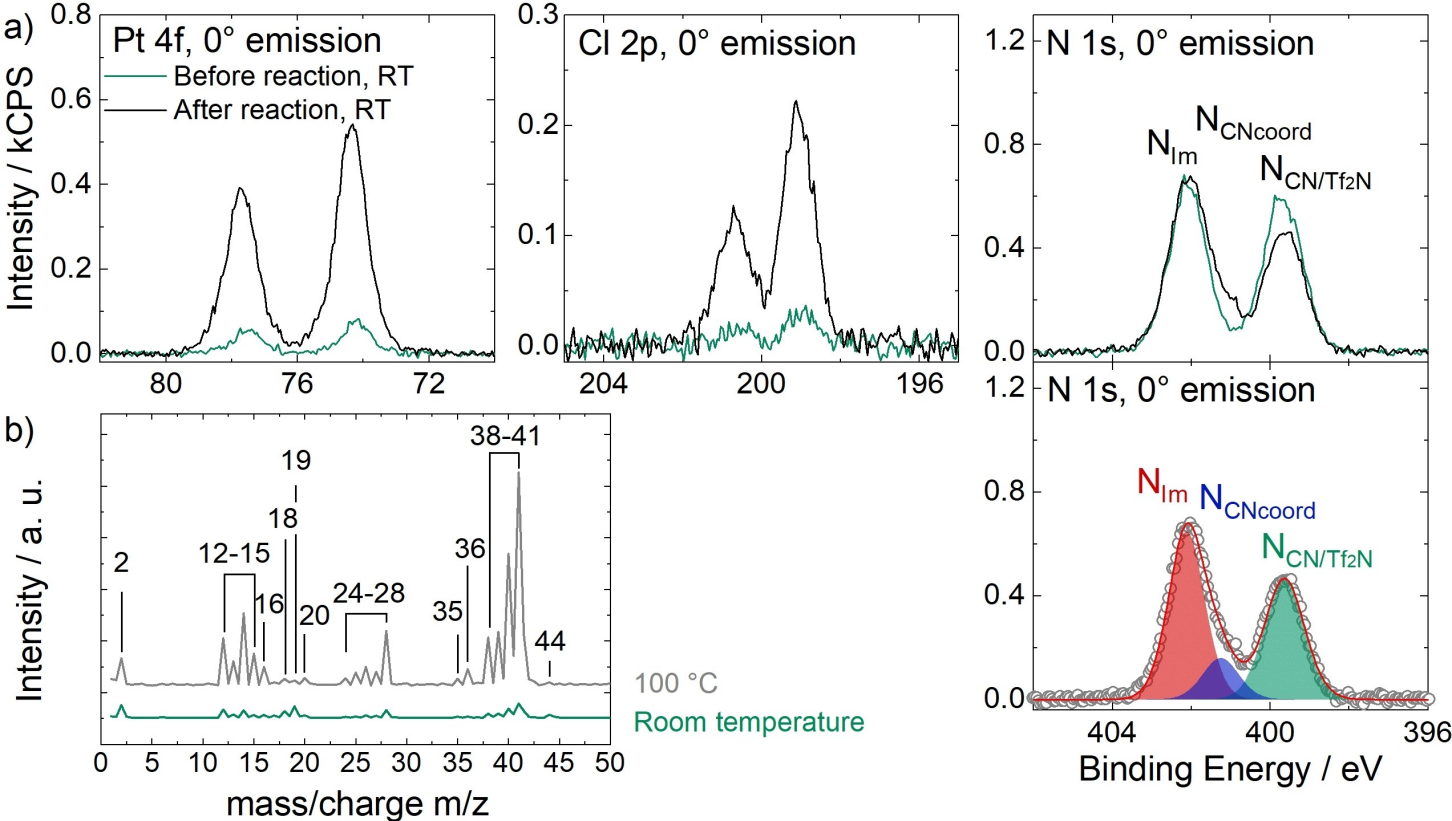
a) Pt 4f, Cl 2p and N 1s XP spectra of a suspension of [PtCl_2_(CH_3_CN)_2_] in [C_3_CNC_1_Im][Tf_2_N] with 1 : 4 molar ratio before (green) and after (black) ligand substitution to yield a clear solution of **1** recorded in 0° emission at room temperature and color‐coded deconvolution applied to the N 1s spectrum after reaction, b) mass spectra of the initial reaction mixture at room temperature (green) and at 100 °C (grey). Adapted from Ref.^[56]^ under CC‐BY‐NC‐ND license.

The formation of the final product **1** was successfully monitored using XPS in 0° emission and QMS, as shown in Figure 5 for a mixture of the Pt‐precursor *cis*‐[PtCl_2_(CH_3_CN)_2_] and the IL [C_3_CNC_1_Im][Tf_2_N] with 1 : 4 molar ratio (due to consumption of the solvent being the ligand source, quantitative formation of the final product yielded a 1 : 2 molar ratio, that is, 33.3 %_mol_ of the **1** in the IL). The metal precursor showed a relatively low solubility in the IL, yielding only a suspension, so that also the XP spectra of the precursor solution at room temperature shown in Figure 5 (green) revealed relatively low intensities of the precursor‐specific Pt 4f and Cl 2p signals at 74.2 and 199.0 eV, respectively.^[56]^ The N 1s region showed two distinct peaks corresponding to the N atoms incorporated into the imidazolium ring, N_Im_, at 402.1 eV and a joint signal from N atoms of uncoordinated CN groups of the IL cation and [Tf_2_N]^−^, N_CN/Tf2N_, at 399.7 eV. Owing to the low solubility of the precursor, N atoms of coordinated CN groups of the CH_3_CN ligands were expected to only show a minor contribution N_CNcoord_ at ~401.3 eV (see discussion below). The mass spectrum of the precursor solution measured at room temperature in Figure 5b (green) revealed small signals at 12–15, 24–28, and 38–41 amu assigned to CH_3_CN vapor,^[80]^ indicating slow abstraction of the labile ligands of the precursor under UHV. Heating the mixture to 100 °C (grey in Figure 5b) resulted in a strong increase of these signals, disclosing a boost of the reaction toward practical rates. Upon progress of the reaction, the CH_3_CN‐specific signals and the overall pressure in the chamber decreased to a minimum (not shown), while solid precursor particles were visibly consumed, eventually yielding a clear solution.^[56]^

The success of the preparation route became evident from comparing XP spectra of the precursor suspension (green) with the fully reacted solution (black) shown in Figure 5, revealing a strong increase of the Pt 4f and Cl 2p signals at 74.4 and 199.1 eV, respectively. The intensity now excellently conformed with the nominal intensity expected from the quantitative formation of **1** (33.3 %_mol_).^[56]^ After the reaction, the N 1s region showed an additional third peak, N_CNcoord_, at 401.3 eV, whose intensity nicely matched the 1 : 2 Pt : N_CNcoord_ ratio expected from the structure of **1**.^[56]^ The applied deconvolution of the N 1s region with color‐coded peaks is also shown in Figure 5a. Since the appearance of this peak was accompanied by an equivalent decline of the N_CN/Tf2N_ signal at 399.6 eV, this effect was assigned to successful coordination of [C_3_CNC_1_Im]^+^ to the metal via the CN groups: upon coordination, the CN group acts as an electron donor resulting in a shift to higher binding energy.^[56]^

Our *in vacuo* preparation route has proven feasible for a variety of complexes in the respective CN‐functionalized ILs (complexes **1**–**3**)^[56]^ or in non‐functionalized ILs, namely in [C_4_C_1_Im][PF_6_] (**4** and **5**),[^53^, ^57^] [C_2_C_1_Im][PF_6_], [C_8_C_1_Im][PF_6_], [C_4_C_1_Im][Tf_2_N] (all **5**)^[60]^ in various concentrations.[^53^, ^56^, ^57^, ^60^] In almost all cases, the reaction was also successful under medium vacuum (MV) conditions using Schlenk techniques, which allowed for stirring of the mixtures and, thus, a more practical and widely applicable preparation protocol (see experimental section for details).^[56]^ For a 33.3 %_mol_ solution of **3** in [C_1_CNC_1_Pip][Tf_2_N], however, UHV conditions were required for successful preparation.^[56]^ Notably, the preparation route was unsuccessful in [C_4_C_1_Im]Cl owing to the competing coordination of Cl^−^ from the solvent rather than the CN‐functionalized ligands.^[60]^

**Figure 6:**
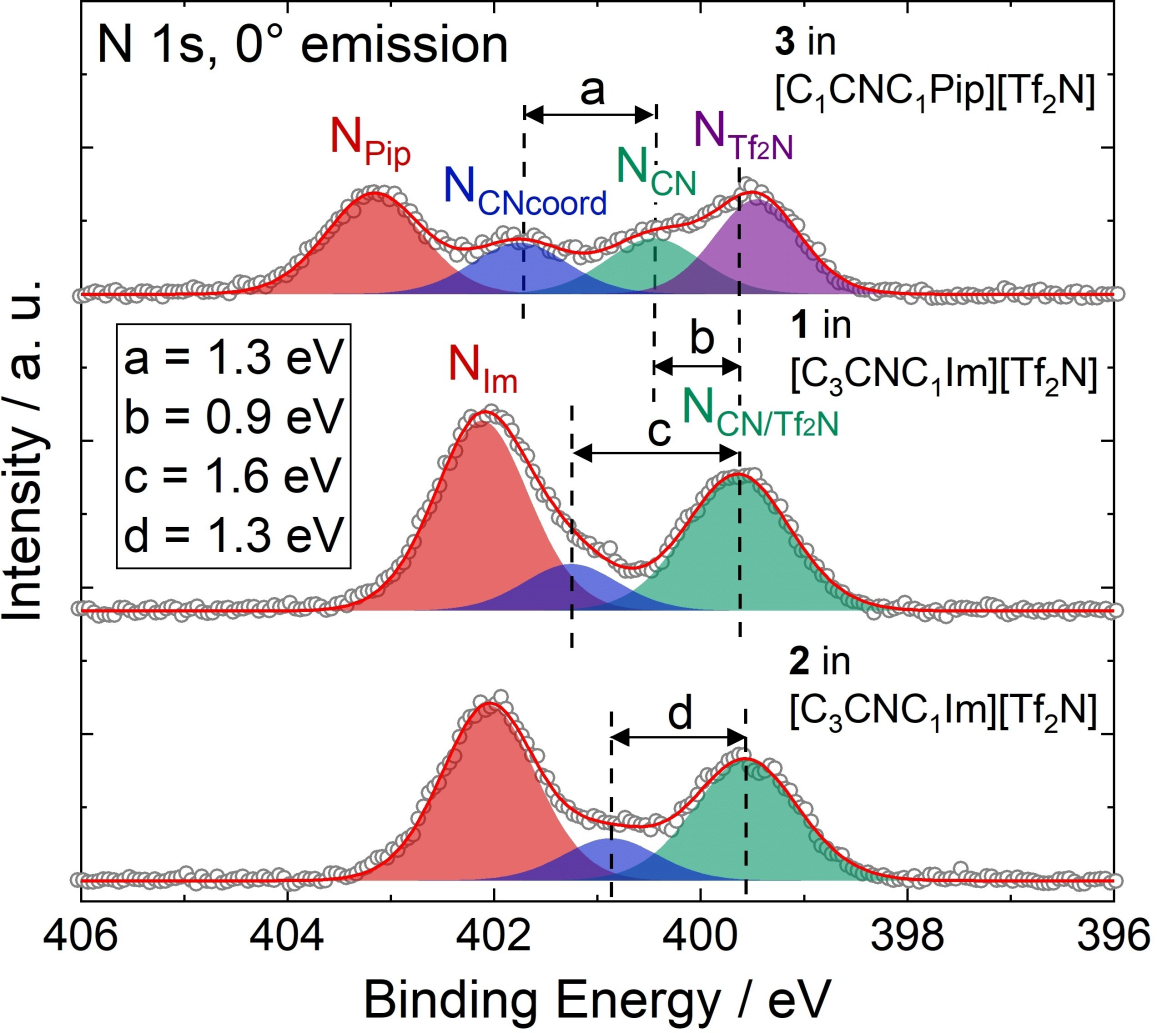
N 1s region spectra of 33.3 %_mol_ solutions of **1** (middle) and **2** in [C_3_CNC_1_Im][Tf_2_N] (bottom), and **3** in [C_1_CNC_1_Pip][Tf_2_N] (top). All spectra were recorded at 0° emission angle at room temperature. Adapted from Ref.^[56]^ under CC‐BY‐NC‐ND license.

The comparison of the XP spectra of complexes **1**–**3** in the respective CN‐functionalized ILs has proven to provide interesting information on the electronic properties of the coordinating moieties and the metal centers. Figure 6 contrasts the N 1s spectra recorded in 0° emission of 33.3 %_mol_ solutions of **1** (middle, see also above) and the Pd analog **2** (bottom) in [C_3_CNC_1_Im][Tf_2_N], as well as the Pt‐piperidinium complex **3** (top) in [C_1_CNC_1_Pip][Tf_2_N]. While for solutions of **1** and **2** the N_CN_ atoms gave a joint signal with the N_Tf2N_ atoms, N_CN/Tf2N_ at 399.6 eV, the N_CN_ signal from the solution of **3** was detected more separated at 400.5 eV and thus shifted about 0.9–1.0 eV (distance *b* in Figure 6) to higher binding energy.^[56]^ This finding indicated a significantly higher electron density at the CN group in the imidazolium derivative [C_3_CNC_1_Im][Tf_2_N] due to the longer separation between the functionalization and the electron‐withdrawing N atoms of the heterocycles, compared to [C_1_CNC_1_Pip][Tf_2_N] with a short separation. This finding was in line with results from a ^15^N NMR study on pyridinium ILs, unveiling that longer CN‐functionalized chains, indeed, result in higher negative charges localized at the nitrile N atoms.^[81]^

These differences in electron density at the coordinating atom, in turn, suggested a stronger coordination power of the [C_3_CNC_1_Im]^+^ cation when compared to [C_1_CNC_1_Pip]^+^: The binding energy difference between the N_CN_ and N_CNcoord_ signals of **1** in [C_3_CNC_1_Im][Tf_2_N] (1.6 eV, distance *c* in see Figure 6, middle) and **3** in [C_1_CNC_1_Pip][Tf_2_N] (1.3 eV, top), induced by coordination of the CN groups to the metal, is about +0.3 eV higher for the long‐chained derivative, which is in line with a stronger donation of electron density to the metal upon coordination. An according shift of the Pt 4f signals by 0.2 eV to lower binding energy for the solution of **1** was also detected, though the magnitude of this shift is at the limit of our experimental uncertainty.^[56]^ Interestingly, the solution of **2** in [C_3_CNC_1_Im][Tf_2_N] also revealed an N_CN_→N_CNcoord_ shift of 1.3 eV, that is, 0.3 eV lower compared to the solution of the Pt analog **1**, and could be assigned to a weaker Pd−N bond than Pt−N found computationally for related systems.^[82]^

In the following passages, the interfacial behavior of the complexes and a successful modification of the ligand system to deliberately maximize the local catalyst concentration at the surface will be discussed along **4** and **5** as model systems in the well‐studied commercially available IL [C_4_C_1_Im][PF_6_]. **4** is equivalent to **1** but with [PF_6_]^−^ as counter anions instead of [Tf_2_N]^−^. The ligand system of **5** was complemented with fluorous butyl chains (PFC_4_), which have exhibited surface activity in binary mixtures of ILs before.[^68^, ^83^, ^84^] The complexes were prepared as [PF_6_]^−^ salts to ensure distinct peaks in the XP spectra for the PFC_4_ chains, which would otherwise superimpose with signals of [Tf_2_N]^−^ also carrying CF_3_ groups.[^53^, ^56^, ^57^, ^60^] Accordingly, the IL was chosen to be an [PF_6_]^−^ IL, and the non‐functionalized butyl chain of [C_4_C_1_Im]^+^ yields a separated, IL‐specific C_alkyl_ signal (see experimental section and discussion below), which allows for extracting the location of the IL cation at the surface.[^53^, ^57^, ^60^]

**Figure 7:**
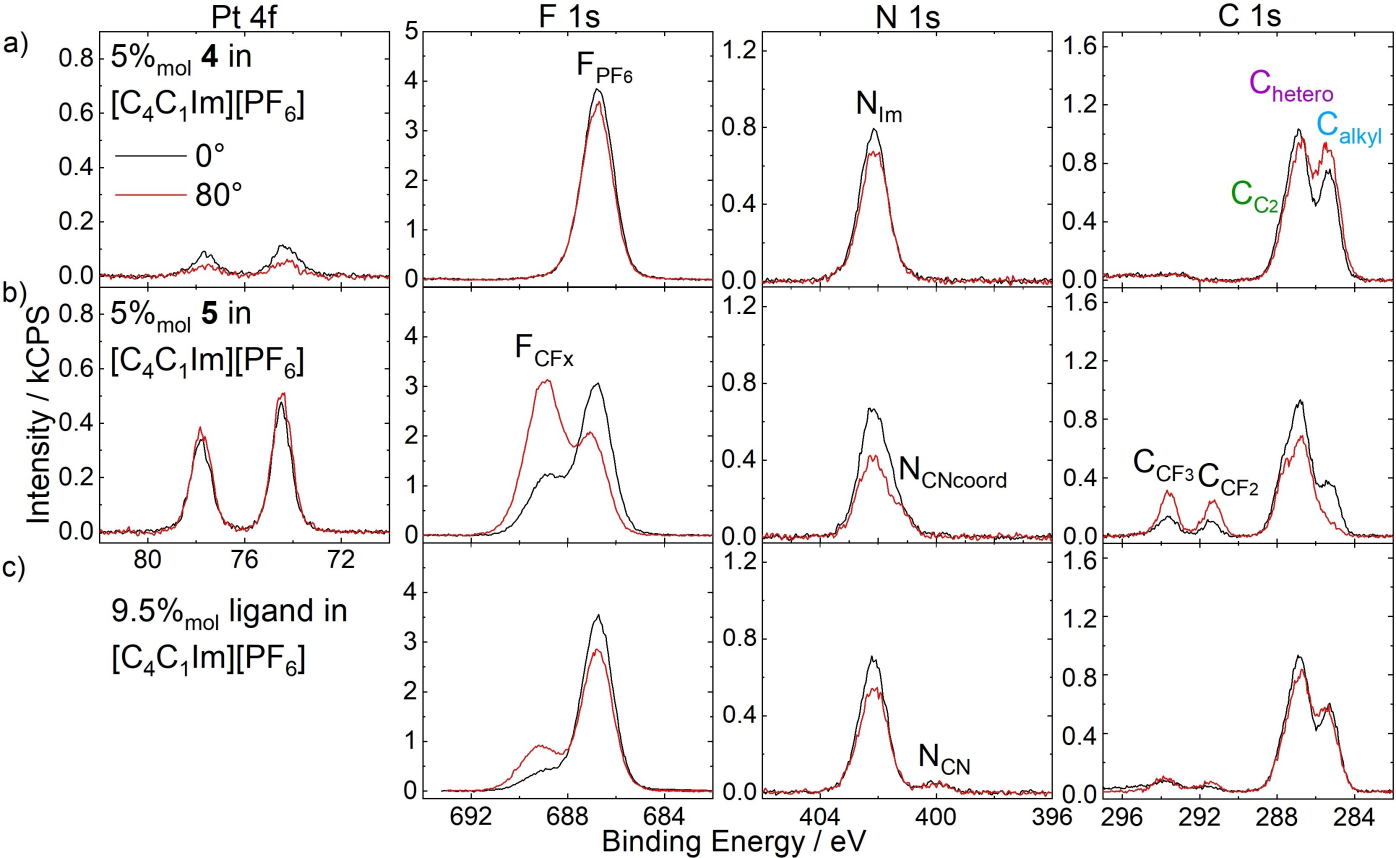
Pt 4f, F 1s, N 1s, and C 1s spectra of 5 %_mol_ solutions of a) **4**, b) **5**, and c) 9.5 %_mol_ solution of [C_3_CNPFC_4_Im][PF_6_] in [C_4_C_1_Im][PF_6_] in 0° (black) and 80° (red) emission. Note that the solutions shown in b) and c) contain the same IL : PFC_4_ ratio of 2 : 19. All spectra were recorded at room temperature. Adapted from Refs.[^53^, ^57^] under CC‐BY‐NC‐ND and CC‐BY‐NC licenses.

Figure 7a depicts the Pt 4f, F 1s, N 1s, and C 1s XP spectra of a 5 %_mol_ solution of **4** in [C_4_C_1_Im][PF_6_] in 0° (black, more bulk‐sensitive) and 80° emission (red, more surface‐sensitive). The Pt 4f_7/2_ signal is detected at 74.3 eV, the F_PF6_ peak at 686.8 eV, and the N_Im_ peak at 402.2 eV originate from both complex and IL. Owing to the relatively low catalyst concentration of 5 %_mol_ and partial overlay with the N_Im_ (cf. Figures 5 and 6), no sufficiently accurate deconvolution of an N_CNcoord_ peak was achieved. Also, no N_CN_ signal (~400 eV, cf. Figures 5 and 6) was detected since the solvent [C_4_C_1_Im][PF_6_] did not contain a CN group. The C 1s region showed a broad envelope typically fitted with three peaks (see experimental section); the assignment of carbon species of complex and solvent can be seen from the color coding in Tables 1 and 2. The intensities detected at 0° excellently agreed with the nominal composition of the solution.^[57]^ At 80°, however, the Pt 4f signals declined to ~50 % of the 0 signals, while the F 1s and N 1s signals showed a slight decrease, and the C_alkyl_ signal significantly increased at 80° by ~30 %.^[57]^ These findings are in line with a surface preferably terminated with the C_4_ chains of the [C_4_C_1_Im]^+^ cation, while the imidazolium rings and [PF_6_]^−^ anions form a polar layer beneath, which is a well‐known effect for ILs with extended alkyl chains, as already discussed above.[^42^, ^43^, ^69^, ^70^, ^71^, ^72^, ^73^, ^74^] The fact that the Pt signal of **4** showed the largest decline at 80° was attributed to a preferential orientation of the homogeneously dissolved complex in the topmost molecular layer, with the charged imidazolium moieties of the ligands incorporated into the imidazolium/[PF_6_]^−^ layer and the Pt center located slightly below this layer.

XP spectra of an equivalent solution of **5**, which only differs from **4** in the PFC_4_ chains, are shown in Figure 7b. The spectra featured additional signals in the F 1s and C 1s regions, F_CFx_ at 688.8, C_CF3_ at 293.7, and C_CF2_ at 291.4 eV, which originate from the PFC_4_ chains. While the F_CFx_ atoms all contribute to a single signal, the fluorinated carbon atoms C_CF3_ and C_CF2_ can be distinguished due to the number of F atoms bound to them.^[68]^ Also, non‐fluorinated C atoms from the PFC_4_ chains contributed to the C_hetero_ signal (see Table 2 for detailed assignment). In addition, the N_CNcoord_ signal at 401.3 eV was now evidently discernible from the N_Im_ signal, showing a much higher intensity. All complex‐specific Pt 4f, F_CFx_, N_CNcoord_, C_CF3_, and C_CF2_ signals showed much higher intensities than the solution of **4**. This effect was even observed in the more bulk‐sensitive 0° measurements. Aside from that, the intensity of the solvent‐specific C_alkyl_ signal at 285.2 eV was detected eminently lower. These observations revealed a strong enrichment of complex **5** at the IL/gas interface and the associated depletion of the [C_4_C_1_Im]^+^ cations. The enrichment is solely caused by the presence of the PFC_4_ chains, which trigger localization of the complex at the interface in a buoy‐like fashion – referred to as the “buoy effect”.^[57]^

More detailed insights into the catalyst‐enriched surface could be extracted from a comparison of 0° and 80° spectra in Figure 7b. All complex‐specific signals increase at 80°, being most prominent for the F_CFx_, C_CF3,_ and C_CF2_ signals of the PFC_4_ chains, while the Pt 4f peaks only showed a weak increase. We assigned this behavior to a preferential surface orientation of **5** with the PFC_4_ chains exposed to the vacuum and the Pt center located beneath. The C_alkyl_ signal showed a drastic decline at 80° to an extent where it is only a small shoulder of the C 1s envelope, stressing the extreme enrichment of **5** and the concomitant depletion of the solvent.^[57]^

Additional information on the buoy effect was elucidated by investigating the surface tension of the two solutions using the pendant drop (PD) method.^[53]^ The surface tension of the catalyst solutions was measured under ultra‐clean vacuum conditions in a newly developed chamber.[^53^, ^67^] At 298 K, the 5 %_mol_ solution of **4** showed a surface tension of 43.9 mN/m, which is even slightly higher than observed for neat [C_4_C_1_Im][PF_6_] with a surface tension of 43.4 mN/m.^[53]^ In contrast, for the 5 %_mol_ solution of **5**, a value of 40.0 mN/m was found at 298 K, which is significantly lower than for the solution of the non‐surface‐active derivative and the neat IL. These observations attested to the lowering in surface free energy, signified by the measured surface tension, as the driving force for the strong accumulation of **5** at the IL/vacuum interface.^[53]^

Given that the strong enrichment of **5** at the IL/vacuum interface was solely induced by the buoy‐like character of the two PFC_4_−functionalized ligands, the interfacial behavior of the ligand as an individual solute without being attached to the metal center was also investigated.^[53]^ For this, a solution with a 9.5 %_mol_ concentration of only the ligand [C_3_CNPFC_4_Im][PF_6_] in [C_4_C_1_Im][PF_6_] was prepared, providing an identical IL : ligand ratio of 2 : 19 as in the 5 %_mol_ solution of **5**; the only difference is the absence of the Cl_2_Pt‐moiety as the coordination partner. The XP spectra shown in Figure 7c revealed much lower intensities for the ligand‐specific signals and a much higher intensity for the IL‐specific C_alkyl_ signal, when compared to the solution of **5** in Figure 7b. The intensities corresponded only to a moderate surface enrichment of the ligand itself in the IL, and, consequently, the surface enrichment of uncoordinated [C_3_CNPFC_4_Im][PF_6_] was found much less pronounced than for **5**, which carries two of these surface‐active ligands. This observation was assigned to the fact that for removing **5** from the IL/vacuum interface and diffusion into the bulk, the simultaneous removal of two surface‐affine PFC_4_ chains must be accomplished, which one might denote a “surface chelate effect”, in analogy to the chelate effect in coordination chemistry.^[53]^ In a broader sense, this result indicated that the catalyst design with two (or even more) surface‐active ligands bound to the metal center rather than one yields a much higher degree of enrichment.^[53]^

**Figure 8:**
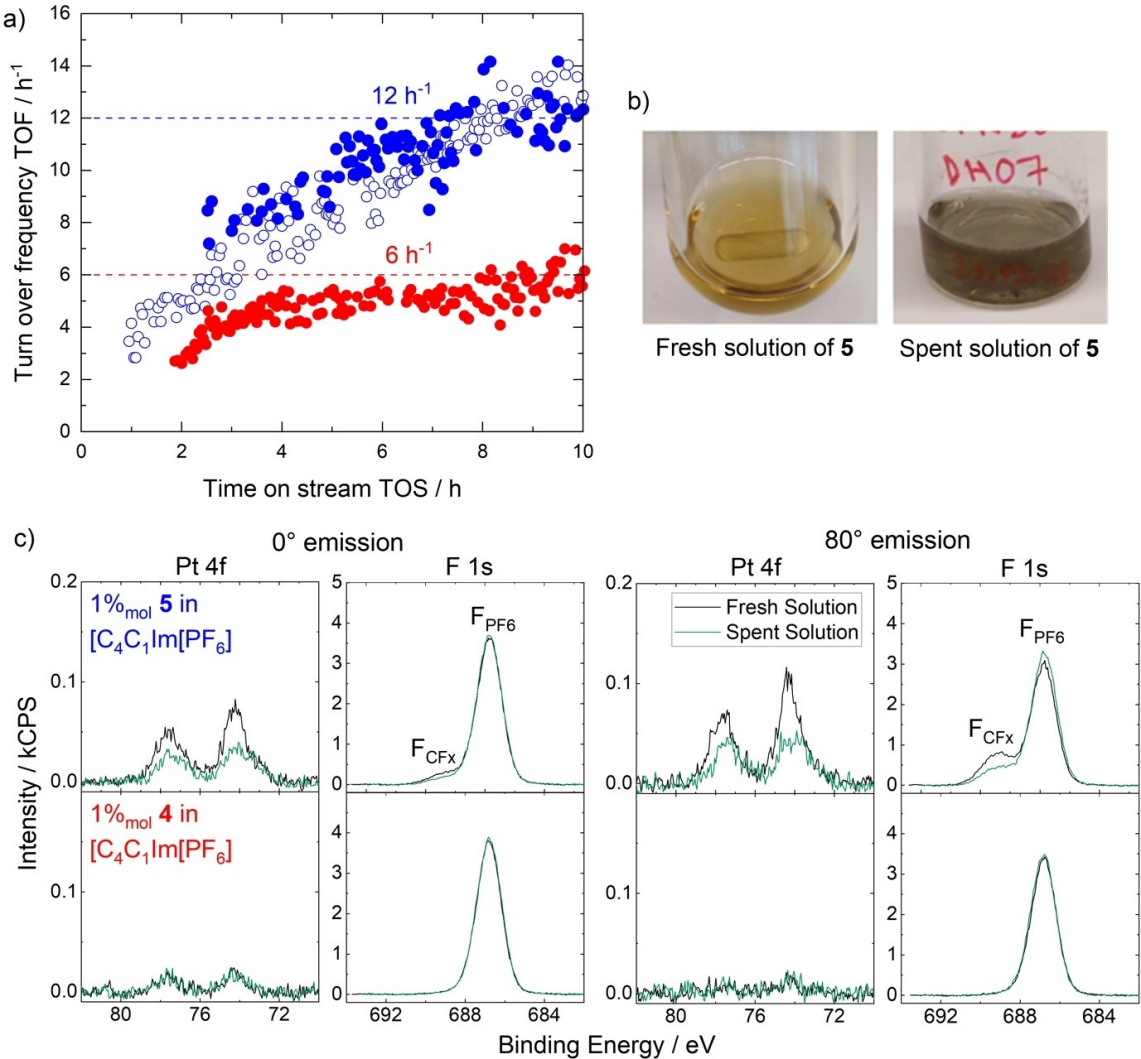
a) Calculated turnover frequency (TOF) values of two runs (open and full symbols) in catalytic hydrogenation with 1 %_mol_ solutions of **4** (red) and **5** (blue) in [C_4_C_1_Im][PF_6_] (reaction conditions: 1.6 ml solution, T=313 K, pressure 0.62 MPa, composition of feed stream: 68 %_vol_ Ar, 16 %_vol_ H_2_, 16 %_vol_ C_2_H_4_: residence time 42 s), b) photographs of fresh and spent (after 10 h at 313 K and catalytic conditions) catalyst solutions of **5**, c) Pt 4f and F 1s XP spectra of fresh (black) and spent (green)1 %_mol_ solutions of **5** (top) and **4** (bottom) in [C_4_C_1_Im][PF_6_] in 0° (left) and 80° emission (right) at room temperature. Reproduced from Ref.^[63]^

With the successfully achieved buoy‐like accumulation of **5** at the IL/vacuum interface and a non‐enriched catalyst **4** suitable for comparison, the impact of the surface enrichment on the catalytic performance was studied for hydrogenation of ethene.^[63]^ The reactor setup involved a pool with a stationary film of IL solution with a well‐defined planar gas‐liquid contact area (71×22.5 mm) to study the impact of the enhanced catalyst concentration at the surface.^[63]^ 0.05 %_mol_ and 1 %_mol_ solutions of **5** and **4** in [C_4_C_1_Im][PF_6_] were investigated and, indeed, the solutions of **5** yielded a two times higher activity, expressed as turnover frequency (TOF) at 313 K after 9 and 10 h time on stream, respectively; the catalytic results for two runs (full and open symbols) of the 1 %_mol_ solutions of **5** (blue) and **4** (red) in [C_4_C_1_Im][PF_6_] are shown in Figure 8a. However, as visible with the naked eye (see Figure 8b) and additionally confirmed by *in situ* light scattering (not shown), metallic Pt particles have formed acting as heterogeneous catalysts under the reductive conditions of the hydrogenation reaction.^[63]^

ARXPS analyses were conducted before and after the hydrogenation experiment for the 1 %_mol_ solutions.^[63]^ Pt 4f and F 1s XP spectra at 0° (left) and 80° (right) of solutions of **5** (top) and **4** (bottom) are shown in Figure 8c. During transfer between the experimental setups used for catalysis and the ARXPS analyses, the relatively large Pt particles settled to the ground of the vessel, and no metallic Pt could be observed in XPS (BE~71.7 eV).^[53]^ Before catalytic conversion, the spectra resemble the characteristics discussed above for the 5 %_mol_ solutions and reflect surface enrichment of **5**, while **4** showed no surface affinity. After the catalytic experiment (green), however, the solution of **5** showed a pronounced decrease in intensity of the complex‐specific Pt 4f and F_CFx_ signals at both 0° and 80°, while the F_PF6_ signal remained constant at 0° and showed a minor increase at 80°. All other core levels (not shown) reflected similar characteristics.^[63]^ These observations were in line with a remaining lower amount of **5** dissolved in solution due to the formation of metallic Pt particles, which are, however, not within the information depth of XPS.^[63]^ The particles are possibly stabilized by surface‐attached N‐heterocyclic carbenes (NHC), at least as transient species.[^85^, ^86^, ^87^] For the solution of **4** (bottom), the signal intensities remain on a steady level before and after the catalytic experiment due to a lower degree of Pt particle formation due to the lower activity of **4**. The higher activity of **5** was assigned to the higher catalyst concentration at the IL/gas interface.^[63]^

### Polypyridyl‐Based Catalysts

Polypyridyl ligands offer a large diversity of structures and are widespread in various organometallic application areas – bipyridine was even denoted as the “most widely used ligand”,^[88]^ at the turn of the millennium. In view of catalysis, polypyridyl complexes raised significant interest in water oxidation,[^89^, ^90^] water gas shift reaction,[^91^, ^92^] and CO_2_ reduction,^[93]^ among more examples.^[94]^ A Ru‐based polypyridyl complex was also investigated for the coordination of CO_2_ in [C_2_C_1_Im][Tf_2_N] to create a formate species.^[95]^

A first set of Ru(II)‐based polypyridyl complexes, **6** and **7**, was investigated in the ILs [C_4_C_1_Im][PF_6_] and [C_2_C_1_Im][OAc].^[64]^ The complexes both contained a tridentate terpyridine (tpy) ligand, and a bidendate bipyridine (bpy) or a dicarboxylated bpy (dcb), respectively, and a Cl ligand. Solutions with a nominal catalyst concentration of 2.5 %_mol_ were prepared. However, only **6** in [C_2_C_1_Im][OAc] showed full dissolution, while **6** in [C_4_C_1_Im][PF_6_] and **7** in [C_2_C_1_Im][OAc] showed acceptable dissolution (for XPS) of around ~1.3 %_mol_, with some remnants of undissolved complex. In [C_4_C_1_Im][PF_6_], **7** was practically insoluble so that no complex‐related XP signals could be observed at all. The ILs were chosen since the more hydrophobic IL [C_4_C_1_Im][PF_6_] exhibits a considerably lower surface tension (43.4 mN/m at 298 K)^[53]^ than the more hydrophilic IL [C_2_C_1_Im][OAc] (47.1 mN/m at 298 K)^[96]^ Moreover, for [C_4_C_1_Im][PF_6_] a widely inert character was expected, while the basic character of [C_2_C_1_Im][OAc] increases the potential for deprotonation of C_C2_ atoms of the [C_2_C_1_Im]^+^ cation forming acetic acid and the respective N‐heterocyclic carbene (NHC) capable of coordination.^[64]^ An NHC‐acetic acid complex and acetic acid were detected in the gas‐phase over [C_2_C_1_Im][OAc] by UPS and MS,^[97]^ but the general presence of NHCs in [OAc]^−^‐based ILs is still under debate.^[98]^

Indeed, our XPS investigations indicated a different chemical behavior of **6** in the two ILs: While in [C_4_C_1_Im][PF_6_], the complex was found chemically intact, the measurements of a [C_2_C_1_Im][OAc] solution indicated a second Ru species formed by ligand substitution, possibly with the coordinating [OAc]^−^ anion or N‐heterocyclic carbenes generated in the IL (under vacuum). In the course of this study, acetic acid vapor, which formed alongside the NHC species, was detected over the neat IL and the catalyst solution by QMS supporting the presence of NHCs in solution, similar to the UPS and MS results by *Hollóczki* et al.^[97]^ Interestingly, this chemical alteration was not observed for **7** in [C_2_C_1_Im][OAc] indicating that the carboxylic acid groups prevented (partial) degradation of the complex in the IL.^[64]^

For all solutions, the complex‐specific signals showed a strong decrease at 80° emission, which could not be explained by a sole orientational effect but unambiguously indicates depletion of **6** and **7** from the IL/vacuum interface.^[64]^ By contrast, the second Ru species found for the solution of **6** in [C_2_C_1_Im][OAc] showed no angular dependency, indicating a different interfacial behavior of this species due to the different ligand spheres formed in the solution.^[64]^

Since overall the non‐functionalized and COOH/COO^−^‐functionalized polypyridyl ligands of **6** and **7**, respectively, did not induce surface enrichment but rather depletion of the complexes from the surface, we conducted a systematic study on a second set of polypyridyl complexes **8**–**11**, where the ligands were functionalized with different side chains to achieve surface segregation.^[65]^ Owing to the environmental and toxicologic concerns of per‐ and polyfluoroalkyl substance (PFAS)‐based side chains,[^99^, ^100^] as used above to facilitate surface enrichment of **5**, and the proposed ban of such substances on the European Union level, we considered only non‐fluorinated modifications.^[65]^

In complexes **8**–**11**, all coordination sites are occupied by bpy ligands: Two were functionalized with carboxylate groups (dcbNa) introducing charges for satisfying solubilities, and one bpy ligand was modified with two C_9_ (in complex **8**), C_1_ (**9**), ethoxy (OC_2_, **10**) or tert‐butyl (t‐C_4_, **11**) groups, acting as the potentially surface‐active moieties. Using long C_9_ alkyl chains for surface enrichment was inspired by a previously published study on a Ru complex with a trioctylphosphine (C_8_ chains) and a *para*‐cymene ligand showing strong enrichment in [C_2_C_1_Im][Tf_2_N] at the IL/vacuum interface, as was derived from XPS, reactive‐atom scattering (RAS) and time‐of‐flight secondary ion mass spectrometry (TOF‐SIMS).^[101]^ However, the authors studied only one complex, and the solution showed a surface‐active polysiloxane contamination, which might have influenced the enrichment effect.^[101]^ Metallosurfactant‐like complexes were also investigated in aqueous solution, showing enrichment with a special focus on structural properties at the water/air interface and shapes of micelles.[^102^, ^103^, ^104^, ^105^]

**Figure 9:**
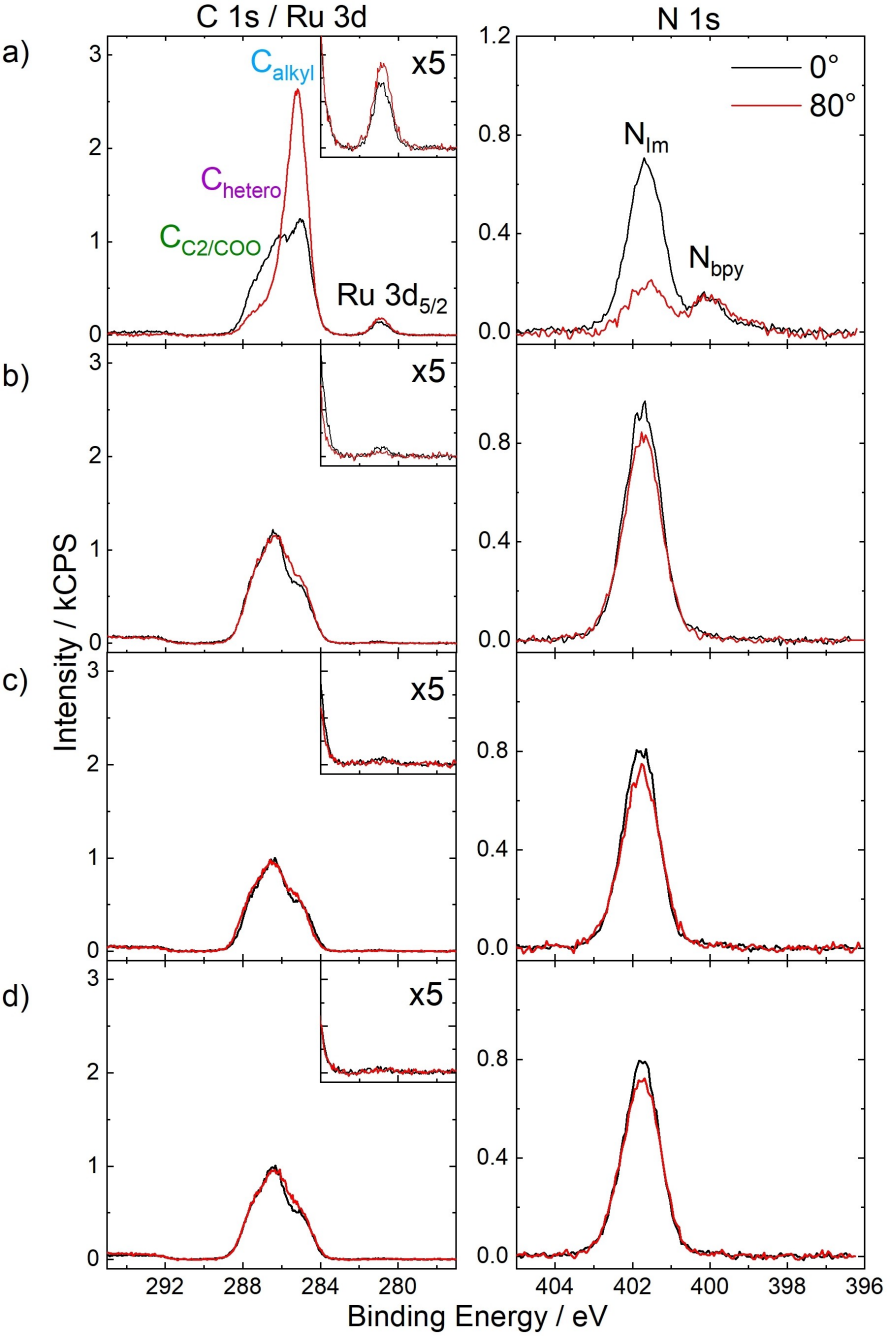
C 1s/Ru 3d and N 1s XP spectra of 1 %_mol_ solutions of a) **8**, b) **9**, c) **10**, and d) **11** in [C_2_C_1_Im][OAc] in 0° (black) and 80° emission (red). Upscaled Ru 3d_5/2_ signals (×5) are depicted in the insets. All spectra were recorded at room temperature. Adapted from Ref.^[65]^ under CC BY license.

1 %_mol_ solutions of **8**–**11** in [C_2_C_1_Im][OAc] visibly displayed full dissolution of the complexes and are discussed in the following to systematically compare the interfacial behavior of the attached organic groups. Figure 9a depicts C 1s/Ru 3d and N 1s XP spectra of the solution of **8**. Very intense Ru 3d_5/2_ and N_bpy_ signals were detected at 280.9 and 400.0 eV, respectively, which are at similar binding energies as found for Ru catalyst solutions discussed above.[^64^, ^65^] The Ru 3d_5/2_ signal corresponded to only one Ru species (see inset for 5x enhanced signal), ruling out a chemical alteration in solution as observed for the solution of **6** in [C_2_C_1_Im][OAc]; also, the Ru 3d : N_bpy_ ratio excellently matched the stoichiometry of the complex confirming stability in solution.[^64^, ^65^] Both the Ru 3d_5/2_ and the N_bpy_ signals showed a largely enhanced intensity at 0° and 80° compared to the nominal composition, which was immediately indicative of pronounced enrichment at the IL/vacuum interface.^[65]^ The C_alkyl_ signal, which contains contributions from the IL and the ligand system of **8**, showed an immense increase at 80°, while the Ru 3d_5/2_ and N_bpy_ signals only slightly increased revealing the C_9_ chains as the surface‐active moieties terminating the surface.^[65]^ According to the strong enrichment of **8**, the solvent IL was found depleted, as is evident from the N_Im_ signal showing a much lower than nominal intensity at 0° and a further strong decrease at 80°. Overall, these results for the Ru complex **8** with two fluorine‐free alkyl chains were in analogy to the buoy‐like enrichment encountered for Pt complex **5** with its PFA‐based side chains, and thus provide an interesting and environmentally less concerning alternative to these systems.^[65]^

Notably, this fluorine‐free buoy effect was not detected for equivalent 1 %_mol_ solutions of the complexes **9**–**11** without long alkyl chains shown in Figures 9b‐*‐*d.^[65]^ The complex‐specific Ru 3d_5/2_ and N_bpy_ signals showed intensities barely discriminable from the background, and the spectra resemble findings discussed for solutions of complexes **6** and **7**, suggesting depletion from the IL/vacuum interface also for **9**–**11** in [C_2_C_1_Im][OAc]. These results thus revealed that the C_1−_, OC_2_ or t‐C_4_‐modified bpy ligands were unsuitable for inducing surface enrichment of the complexes in this IL.^[65]^

### Bis(NHC) Catalyst

Inspired by the strong enrichment of **8** in [C_2_C_1_Im][OAc], which was achieved through functionalization of the bpy ligands with C_9_ chains, we synthesized a Pt complex carrying two NHC ligands modified with a C_8_ chain, and characterized its interfacial behavior.^[54]^ This investigation intended to provide a system suitable for hydrogenation reactions, which remains a homogeneous catalyst under typical reaction conditions, other than complexes **4** and **5**, as discussed above.^[54]^ The NHC‐ligands were also functionalized with a mPEG_3_ chain to ensure satisfying solubilities in PEG‐functionalized ILs, which have attracted significant attention as solvents and electrolytes in the recent past.[^58^, ^106^, ^107^, ^108^] It has been shown that incorporating ether and ester functionalities into the molecular structure of ILs can result in an enhanced biodegradability and lower toxicity,[^109^, ^110^] and interesting physiochemical properties, such as relatively low viscosities.[^110^, ^111^] Bis‐PEG‐ILs, including [(PEG_2_)_2_Im] I, which was one of the solvents used here, had been object of recent ARXPS and PD investigations in our group.[^58^, ^67^, ^112^] These studies on the neat ILs revealed a preferential termination of the IL/vacuum interface with the PEG‐functionalized chains.[^58^, ^112^]

**Figure 10:**
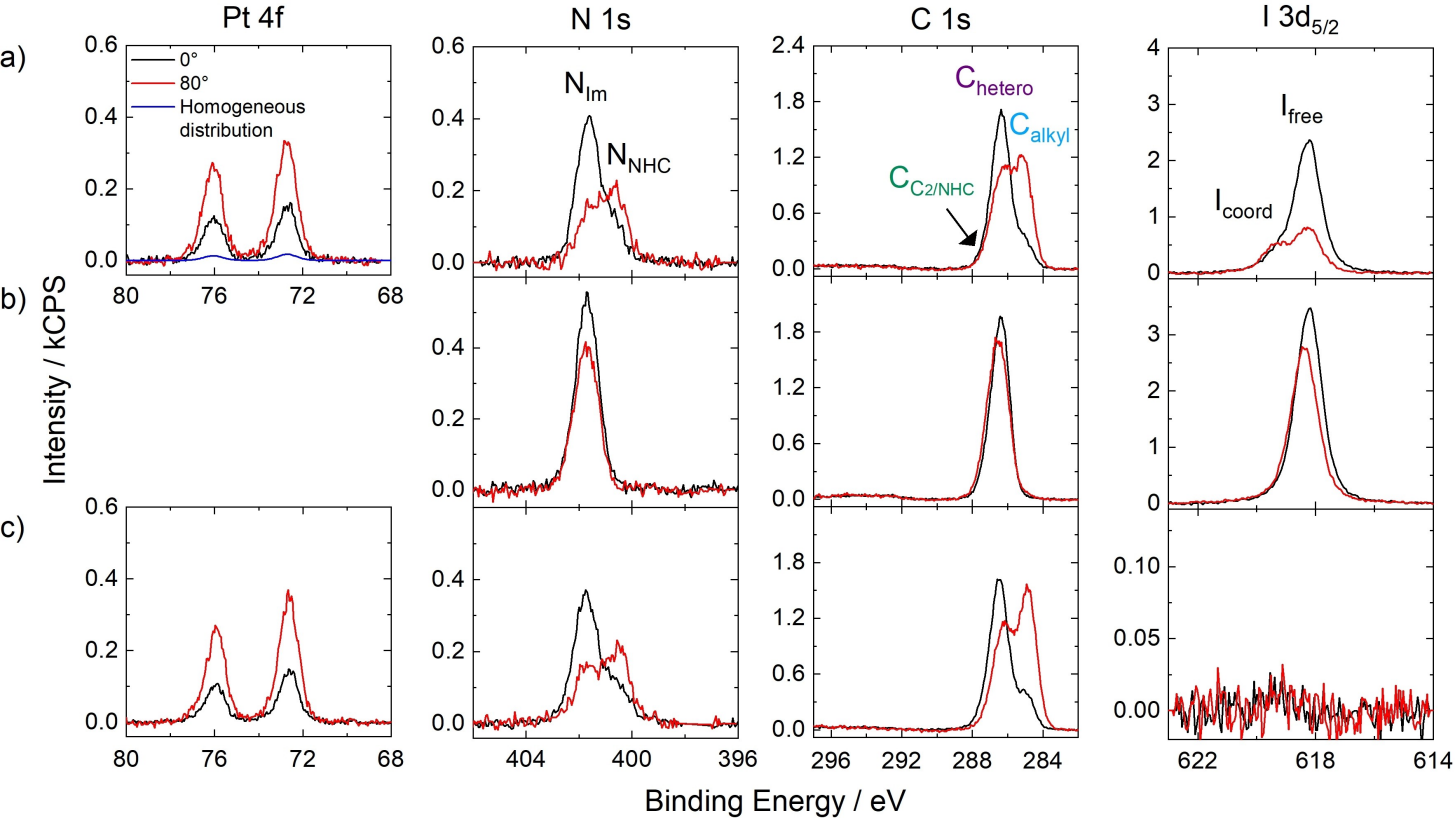
Pt 4f, N 1s, C 1s, and I 3d_5/2_ XP spectra of 1 %_mol_ solutions of **12** in a) [(mPEG_2_)_2_Im] I and c) [(mPEG_2_)_2_Im][PF_6_]. b) N 1s, C 1s, I 3d_5/2_ XP spectra of pure [(mPEG_2_)_2_Im] I. The XP spectra were recorded in 0° (black) and 80° emission (red) at room temperature; in addition, the Pt 4f intensity expected for homogeneous distribution at the surface, that is, without surface enrichment of the complex, is indicated in a) (blue). Reproduced from Ref.^[54]^ under CC‐BY‐NC‐ND license.

Figure 10a shows the Pt 4f, N 1s, C 1s and I 3d_5/2_ ARXP spectra of a 1 %_mol_ solution of **12** in [(PEG_2_)_2_Im] I. For clear identification of the complex‐related signals, we also show the corresponding spectra of neat [(PEG_2_)_2_Im] I in Figure 10b. The spin‐orbit‐resolved Pt 4f_5/2_ and 4f_7/2_ peaks of **12** were detected at 76.1 and 72.7 eV, respectively. Besides the major N_Im_ signal at 401.7 eV, a discernible low‐binding energy shoulder N_NHC_ was detected at 400.6 eV in the N 1s region, corresponding to the N atoms of the NHC ligands.^[54]^ The excellent match of the Pt:N_NHC_ ratio of 1.0 : 4.3 derived from the 0° spectra indicated the intactness of the Pt‐NHC coordination in solution.^[54]^ In the C 1s region, the C_C2_ atoms of the IL and the coordinated C_NHC_ atoms of the NHC ligands were treated as a joint signal, C_C2/NHC_, at 287.3 eV; the C_hetero_ atoms from **12** and the IL yielded a peak at 286.4 eV, and the C_alkyl_ signal at 285.0 eV was only due to the alkyl chains in **12**.^[54]^

The I 3d_5/2_ region is dominated by a major peak, I_free_, at 618.2 eV, which was also observed for the neat IL, and was assigned to free I^−^ anions. The additional unexpected high‐binding energy shoulder at 619.4 eV, I_coord_, indicated a non‐innocent character of the IL by at least partial coordination of I^−^ anions to the Pt center, possibly by substituting a Cl ligand.^[54]^ The latter assumption was driven by a deficiency of Cl ligands derived from the Pt : Cl ratio of only 1.0 : 1.3 at 0° (instead of nominally 1 : 2).^[54]^ However, NMR studies revealed a Pt‐containing byproduct from synthesis differing from the stoichiometry of **12**, which was not identified using ARXPS but might have influenced the Pt : Cl ratio.^[54]^ Synthesis of an intensively cleaned batch without this second Pt species resulted in a Pt : Cl ratio only slightly below the nominal 1 : 2 ratio; this batch, however, was not suitable for discussion of the surface composition due to a surface‐active Si contamination.^[54]^ The non‐innocent character of the I^−^ IL was further underlined by the investigation of an equivalent solution of **12** in [(PEG_2_)_2_Im][PF_6_], which did not show any I 3d_5/2_ peak, and we thus excluded that the I_coord_ peak was due to a contamination from synthesis.^[54]^

As was also observed for all the surface‐active complexes **5** and **8**, the complex‐specific Pt 4f, N_NHC_, and C_alkyl_ signals showed much higher intensities at 0° than expected from the nominal composition of the solution; the signals increased even further at 80°, with the strongest increase observed for the C_alkyl_ signal of the C_8_ chains; this observation unequivocally confirms the (PFA‐free) buoy effect also for **12**. As evident from the N_Im_ and I_free_ signals, the solvent ions were highly depleted from the surface. The strong enrichment of the complex is also indicated in Figure 10a, where the expected Pt 4f intensity (blue curve) is much lower than the experimentally obtained one at 0° (black).^[54]^ The PEG_3_ chains were found directed towards the bulk with no presence to the outer surface, even though the surface termination of such PEG chains in neat PEG‐ILs indicated moderate surface activity.^[54]^ The C_8_ chains, however, exhibit a much stronger surface activity,^[54]^ as was also observed in a binary mixture of [(PEG_2_)_2_Im]I and [C_8_C_1_Im][PF_6_], where [C_8_C_1_Im]^+^ cations were found highly surface‐enriched.^[112]^

Notably, in [(PEG_2_)_2_Im][PF_6_], **12** was found enriched to a similar extent as in [(PEG_2_)_2_Im]I, as deduced from the almost identical XP spectra in Figures 10a and *10c*.^[54]^ This observation is in line with the similar surface tension of the two ILs (46.7 mN/m for [(mPEG_2_)_2_Im]I and 45.6 mN/m for [(mPEG_2_)_2_Im][PF_6_], at 293 K);^[59]^ the surface tension is known to influence the local concentration of the catalyst at the interface, as will be discussed below.^[60]^

The results reviewed up to this point provided information on the chemical nature, orientation, and enrichment effects of catalysts with different ligand systems, which had been chosen to enhance the catalyst concentration at the IL/vacuum interface. Apart from structural features of the ligands, surface enrichment effects are expected to be highly affected also by the surroundings of the catalyst and external conditions.[^37^, ^53^, ^57^, ^60^, ^65^] In the following chapters, we will discuss the influence of the bulk concentration of the catalyst, the sample temperature, and the IL solvent, mainly along solutions of the surface‐active complexes **5** and **8**.

## Influence of the Bulk Concentration

**Figure 11:**
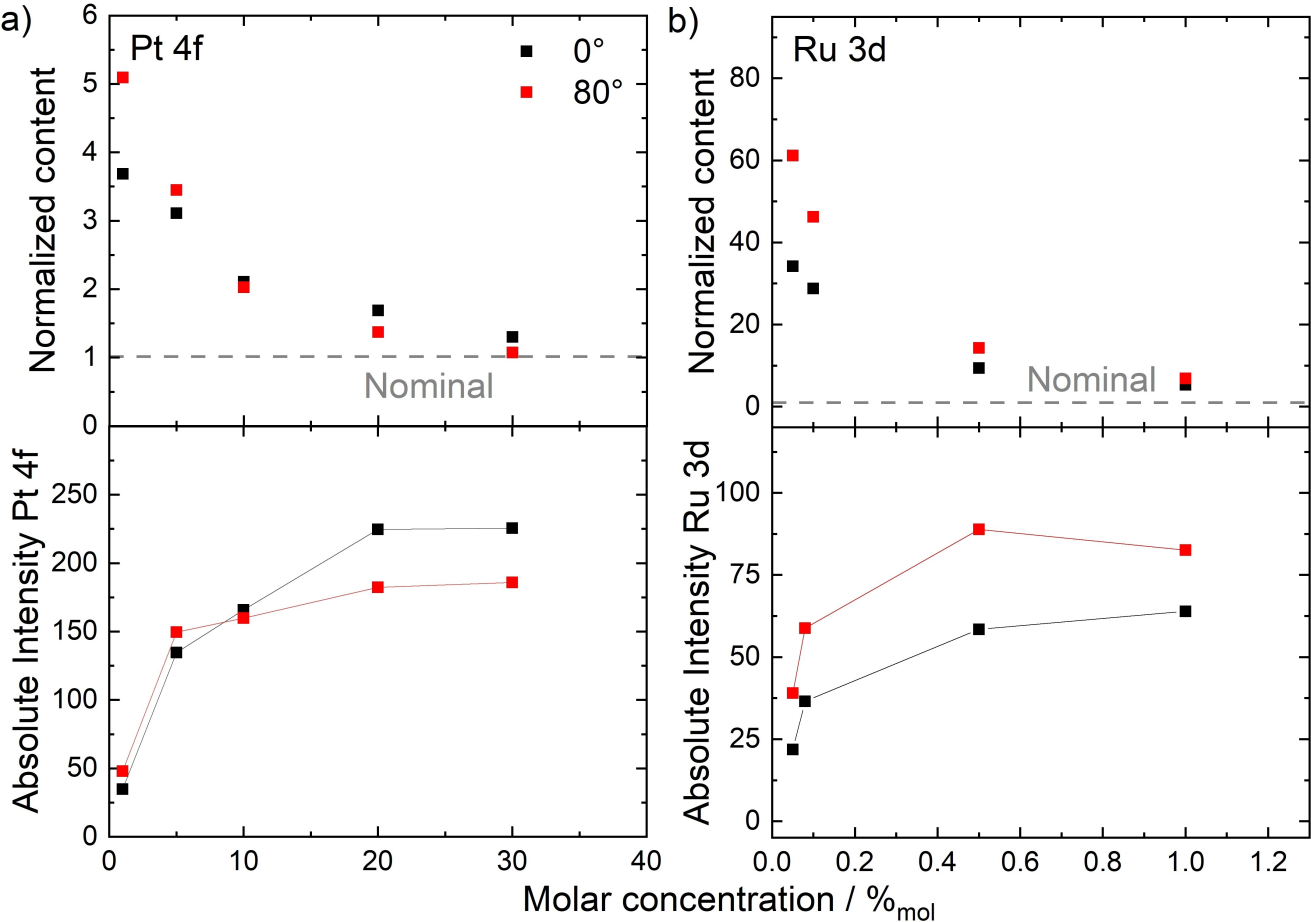
Normalized contents (top) and total peak intensities (bottom) derived from metal signals of a) **5** in [C_4_C_1_Im][PF_6_], and b) **8** in [C_2_C_1_Im][OAc], in 0° (black) and 80° emission (red) upon varying the catalyst bulk concentration. Adapted from Refs.[^53^, ^57^, ^65^] under CC‐BY‐NC‐ND, CC‐BY‐NC, and CC BY licenses.

Owing to the excellent solubility of **5** in ILs, its interfacial behavior was studied over a wide concentration range of 1–30 %_mol_ in [C_4_C_1_Im][PF_6_], which is the solvent used above for demonstrating the buoy effect for **5**.[^53^, ^57^] In Figure 11a, the normalized Pt content is plotted (top) against the molar concentration. The normalized Pt content is defined as the detected Pt 4f signal intensity divided by the nominally expected one; it thus is a measure of the surface enrichment: A value of 1 (indicated by a grey dashed line) corresponds to a situation with homogeneous distribution of the catalyst in the bulk and at the surface, and random surface orientations and configurations of the molecules.[^53^, ^57^] In Figure 11a, both at 0° (black) and 80° emission (red), the normalized content increased upon decreasing the bulk concentration of the catalyst, revealing the most pronounced surface enrichment of the catalyst at the lowest concentration.[^53^, ^57^] This result is greatly promising for catalytic applications, where low catalyst concentrations are practical, with the most efficient metal utilization using a minimum of catalyst in the bulk at maximized surface concentration. A similar trend, namely highest surface enrichment at low concentrations, was found previously for binary mixtures of the structurally related IL [PFC_4_C_1_Im][PF_6_] mixed with [(C_1_O)_2_Im][PF_6_]^[83]^ or [C_4_C_1_Im][PF_6_].^[68]^

Inspection of the absolute Pt 4f intensities shown in Figure 11a (bottom) provided more detailed insights into the surface composition upon varying the catalyst concentration.[^53^, ^57^] While at 0° (black), the intensities increased with increasing concentration according to the higher bulk content of the catalyst up to 20 %_mol_, the 80° intensities (red) only showed a strong initial increase from 1 to 5 %_mol_ but remain more or less on a steady level at higher concentrations (note that the slight increase of the 80° data going from 5 %_mol_ to higher concentrations was assigned to the fact that the (small) bulk contribution to the 80° signal bulk increases with increasing Pt content).[^53^, ^57^] These results suggested that the surface layer is already in the saturation regime at 5 %_mol_ while a concentration of 1 %_mol_ is insufficient to achieve saturation.[^53^, ^57^]

As is visible from Figure 11b, the variation of the concentration of the PFA‐free Ru complex **8** in [C_2_C_1_Im][OAc] yielded a very similar behavior as found for **5** in [C_4_C_1_Im][PF_6_]. For the Ru‐based system, it was even possible to study particularly low concentrations ranging from 0.05 %_mol_ to 1 %_mol_ (mind the different concentration and normalized content scales in Figures 11a and *b*):^[65]^ The enrichment (normalized content) increased strongly upon lowering the concentration (Figure 11b, top), and the absolute Ru 3d intensities at 80° (Figure 11b, bottom, red) showed a plateau starting at 0.5 %_mol_, indicating saturation of the surface with **8** already at 0.5 %_mol_, which is much lower than found for the Pt complex **5** (saturation at 5 %_mol_). We correlate this effect again with the surface tension of the different solvents ([C_4_C_1_Im][PF_6_]: 43.4 mN/m under vacuum^[53]^ and [C_2_C_1_Im][OAc]: 47.1 mN/m; at 298 K),^[96]^ which has shown to substantially affect the surface affinity of solutes in solution, as will be discussed later in this review. However, the structure of the complexes could also contribute to the saturation concentration, influencing the packing density of complexes at the IL/vacuum interface.^[65]^

**Figure 12:**
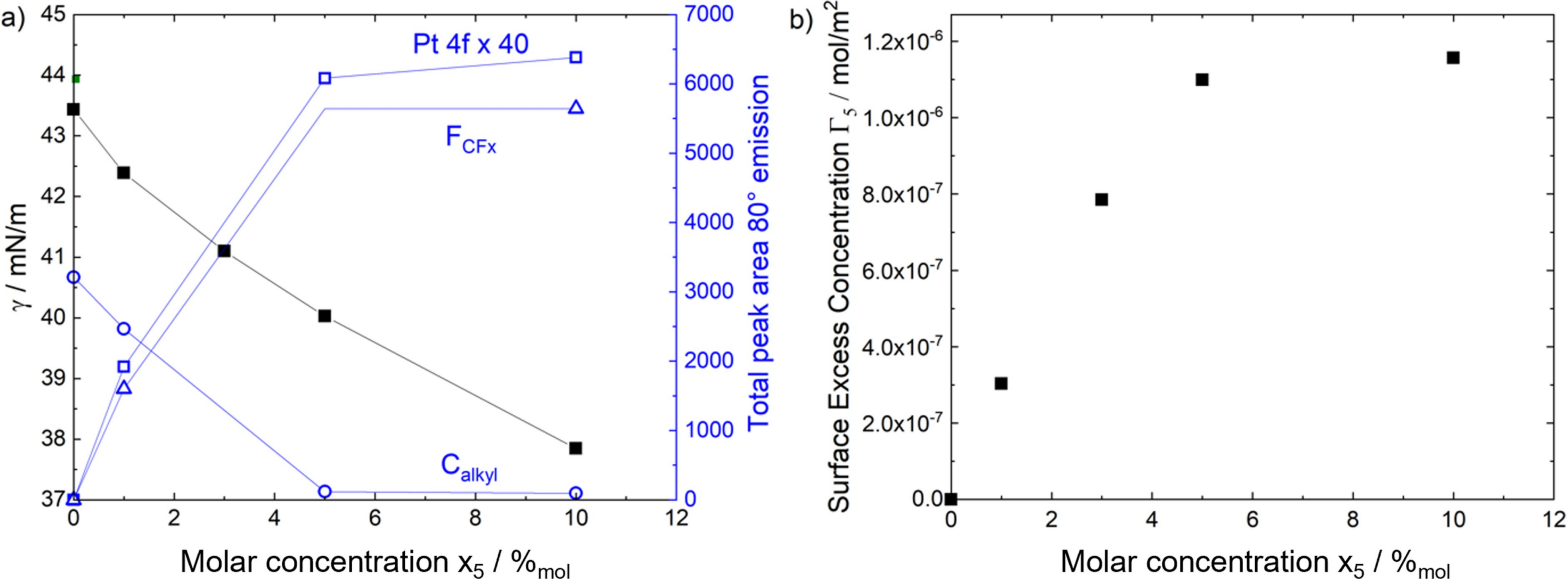
a) Surface tension γ at 298 K (black full squares, referring to left vertical axis) and total peak areas of the Pt 4f (blue open squares, ×40), F_CFx_ (blue open triangles) and C_alkyl_ signals (blue open circles) in 80° emission at room temperature (referring to right vertical axis) of solutions of **5** in [C_4_C_1_Im][PF_6_] over a concentration range of 0–10 %_mol_; adapted from Ref.^[53]^ under CC‐BY‐NC license. b) Surface excess concentration of **5** Γ_5_ calculated from the Gibbs adsorption isotherm vs the molar concentration of **5** x_5_.

The concentration‐dependent enrichment effects extracted from XPS were also correlated to the surface tension of the solutions, which was determined using the PD method. Figure 12a depicts the obtained surface tensions for solutions of **5** in [C_4_C_1_Im][PF_6_] with a catalyst concentration between 0–10 %_mol_ at 298 K (black; left vertical axis) along with the total Pt 4f peak area detected in XPS signals at 80° (blue open squares, right vertical axis) against the molar catalyst concentration.^[53]^ Aside from the Pt 4f signal, the XPS data was now complemented with the F_CFx_ (blue open triangles) and C_alkyl_ (blue open circles) signals.^[53]^ In accordance with the Pt 4f signal, these signals show an increase and a decrease with increasing concentration, respectively, until a plateau is reached at 5 %_mol_; this behavior again emphasized surface saturation with the Pt complex at 5 %_mol_ or higher.[^53^, ^57^] The surface tension values (black solid squares), however, showed a steady decrease with increasing concentration without displaying a plateau at 5 and 10 %_mol_, even though XPS evidenced a similar composition of the topmost surface layer.[^53^, ^57^] This behavior was attributed to a significant change in cohesive forces upon changing the catalyst concentration in the layers below the topmost surface layer (in the bulk), which corresponds to a change in surface tension and is also known for aqueous surfactant solutions where the surface tension decreases beyond saturation until the critical micelle concentration was reached.[^53^, ^113^] Even though the microscopic surface composition of IL mixtures derived from ARXPS was nicely correlated to surface tension data for IL mixtures in the recent past,[^84^, ^112^, ^114^] a complete representation of the ARXPS data with the surface tension was not achieved so far for the system investigated herein.[^53^, ^57^]

The Gibbs adsorption isotherm (see *Equation 1*), however, allows for analysis of the surface excess concentration, which is the deviation of the number of surface‐active molecules within the surface plane from the number of surface‐active molecules nominally located within an equal plane in the bulk. The surface excess concentration of dilute solutions can be derived in a simplified form from the concentration‐dependent surface tension curve at a certain temperature according to^[115]^

(1) $$\Gamma_{5}=\frac{-1}{RT}{\left(\frac{d\gamma }{d{lnx}_{5}}\right)}_{p,T}=\frac{-x_{5}}{RT}{\left(\frac{d\gamma }{dx_{5}}\right)}_{p,T}$$

where *Γ_5_* is the surface excess concentration of **5**, *R* is the universal gas constant, *T* is the temperature, *γ* is the measured surface tension at a given temperature, and *x_5_* is the molar bulk concentration of **5**. The surface tension at 298 K vs molar concentration plot was fitted using a second‐order polynomial fit to calculate the surface excess concentration given in Figure 12b. The surface excess concentration rises with increasing concentration up to a plateau around 1.1–1.2×10^−6^ mol/m^2^ (which corresponds to an area of around 1.5–1.4 nm^2^/molecule) starting at 5 %_mol_, just like the measured absolute intensity of the complex‐specific Pt 4f and F_CFx_ signals in XPS in Figure 12a. This finding emphasized the previously drawn conclusion of a saturated surface starting at 5 %_mol_, while the saturation is not achieved at 3 and 1 %_mol_. These results signify that the surface excess concentration can be nicely correlated to the surface composition derived from XPS.

## Influence of the Temperature

Since homogeneous catalysis can happen over a wide range of temperatures, we also address the thermal behavior of the local catalyst concentration at the surface.^[53]^ The thermal stability of **5** and the wide liquid window of the respective [C_4_C_1_Im][PF_6_] solutions have proven highly suitable for this intention.^[53]^ In this chapter, we will first discuss the results obtained from a 1 %_mol_ solution corresponding to a situation where the surface was not saturated with the complex at room temperature (see previous chapter), and thereafter compare to a solution within the saturation range, that is, 5 %_mol_.

**Figure 13:**
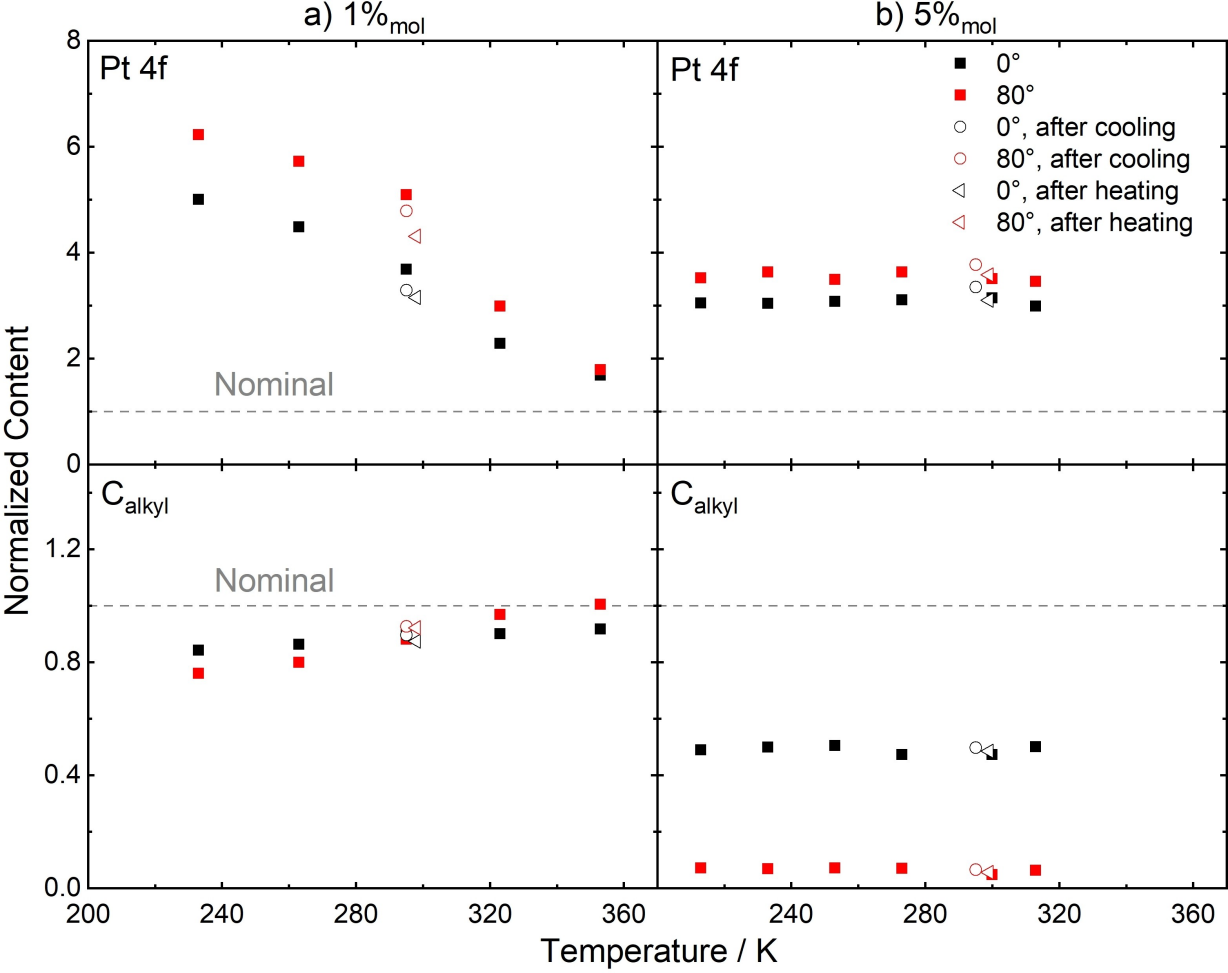
Normalized contents derived from Pt 4f (top) and C_alkyl_ (bottom) signals of a) a 1 %_mol_ and b) a 5 %_mol_ solution of **5** in [C_4_C_1_Im][PF_6_] in 0° (black squares) and 80° emission (red squares) at different temperatures. After measurements at the temperature extremes of the measurement series, the sample was brought back to room temperature and measured again (open circles after cooling series and open triangles after heating series). Adapted from Ref.^[53]^ under CC‐BY‐NC license.

Figure 13a depicts the normalized Pt and C_alkyl_ contents recorded in 0° (black) and 80° (red) from a 1 %_mol_ solution within a temperature range from 233 to 353 K. Both data sets unambiguously reflected a decrease of the Pt content upon raising the temperature, while the IL‐specific C_alkyl_ content increased.^[53]^ These findings corresponded to a lower degree of catalyst enrichment at the surface at higher temperatures; subsequent recording of XP spectra at room temperature after measurements at the temperature extrema (open circles and triangles in Figure 13a) revealed reversibility of the thermal effect.^[53]^ With this, the temperature represents an interesting parameter for simple, sensitive, and reversible adjustment of the surface catalyst concentration. An according temperature dependence was reported before for a binary mixture of the fluorinated IL [PFC_4_C_1_Im][PF_6_] in [C_4_C_1_Im][PF_6_] and was assigned to the larger contribution of the entropic term −TΔS to the surface free energy, favoring a less ordered, that is, a less catalyst‐enriched surface for the solutions.^[68]^

In contrast to the behavior observed for low concentrations, temperature‐dependent measurements of a 5 %_mol_ solution of **5** in [C_4_C_1_Im][PF_6_] over a temperature range from 213 to 313 K, shown in Figure 13b, revealed virtually constant normalized contents at all temperatures measured. With this, no temperature effect was observed, and the saturation of the surface with the complex was maintained over the entire temperature range. It is worth noting that the upper extreme of the temperature series was limited by beam damage; it is well possible that at higher temperatures, the surface saturation could have been disrupted by the thermal effect observed for the 1 %_mol_ solution.^[53]^

## Influence of the Solvent Ionic Liquid

The final parameter affecting surface enrichment phenomena discussed herein is the IL solvent. While the influence of the IL on the electronic properties of organometallics was addressed several times using XPS,[^46^, ^48^, ^116^] the different interfacial composition in solutions of organometallic catalysts when varying the solvent IL was, to the best of our knowledge, only very recently investigated in our group. Solutions of the surface‐active catalyst **5** have proven well‐applicable for this intention,^[60]^ which will be discussed primarily in this chapter besides complementary results from solutions of **12**.^[37]^

**5** was prepared in [C_2_C_1_Im][PF_6_], [C_4_C_1_Im][PF_6_], [C_8_C_1_Im][PF_6_], and [C_4_C_1_Im][Tf_2_N] and found completely soluble in these ILs at all concentrations discussed in the following. The [PF_6_]^−^ ILs only varied in their alkyl chain length *n* of the [C_n_C_1_Im]^+^ cation and, additionally, [C_4_C_1_Im][Tf_2_N] was used to extract the influence of the anion on the surface enrichment compared to [C_4_C_1_Im][PF_6_] sharing the same cation. To broaden the dataset with another anion, [C_4_C_1_Im]Cl was used as the solvent for only the ligand [C_3_CNPFC_4_Im][PF_6_]; this system was compared with the ligand in [C_4_C_1_Im][PF_6_], since preparation of **5** in [C_4_C_1_Im]Cl was not successful, as outlined above.^[60]^

**Figure 14:**
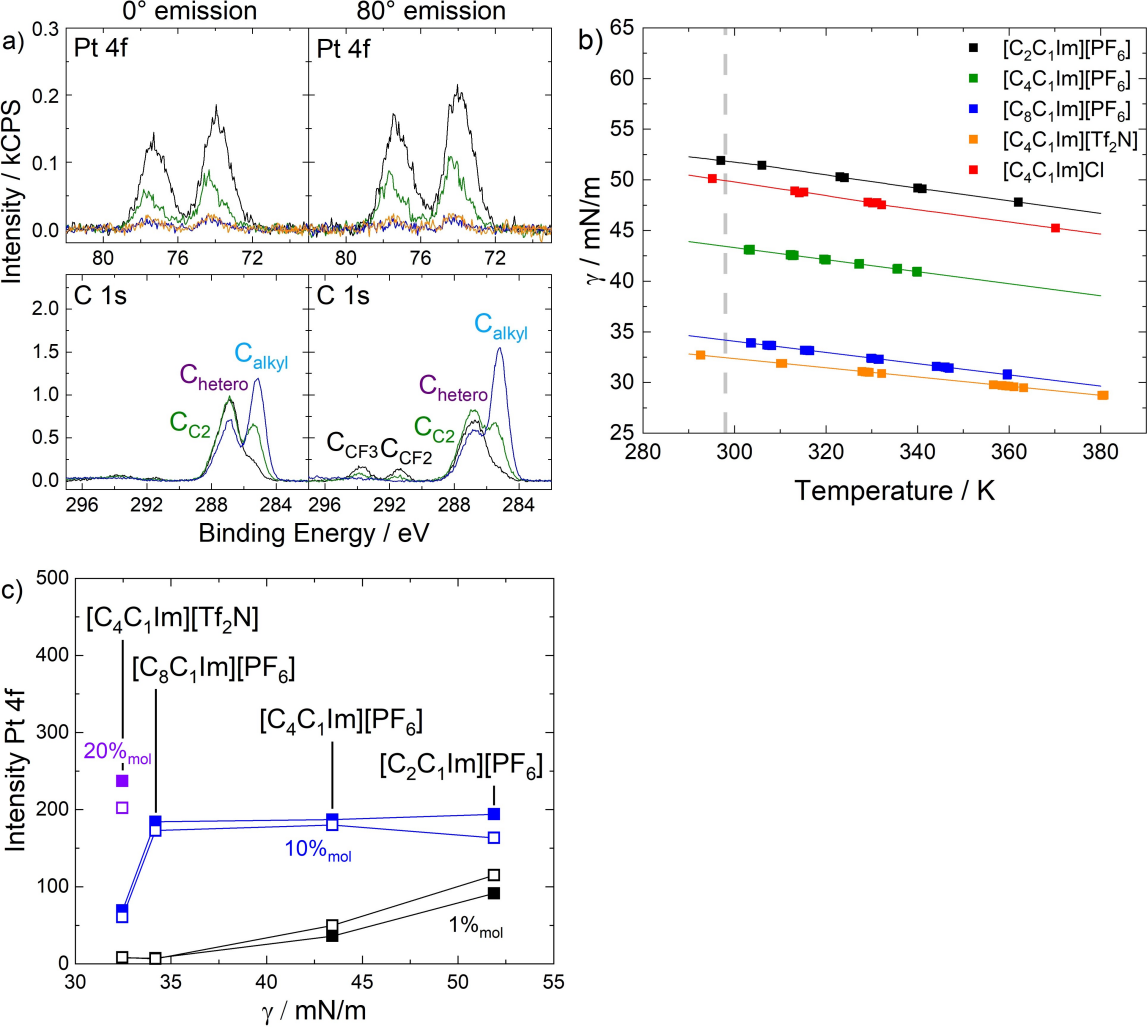
a) Pt 4f (top) and C 1s (bottom) XP spectra of 1 %_mol_ solutions of **5** in [C_2_C_1_Im][PF_6_] (black), [C_4_C_1_Im][PF_6_] (green) and [C_8_C_1_Im][PF_6_] (blue) in 0° (left) and 80° emission (right) at room temperature and Pt 4f spectrum of a 1 %_mol_ solution of **5** in [C_4_C_1_Im][Tf_2_N] (orange), b) temperature‐dependent surface tension γ of neat [C_2_C_1_Im][PF_6_] (black), [C_4_C_1_Im][PF_6_] (green), [C_8_C_1_Im][PF_6_] (blue), [C_4_C_1_Im][Tf_2_N] (orange) and [C_4_C_1_Im]Cl (red), the (grey) dashed line corresponds to 298 K, c) absolute Pt 4f intensity from solutions of **5** in the ILs with concentrations of 1 %_mol_ (black), 10 %_mol_ (blue) and 20 %_mol_ (violet) in 0° (full symbols) and 80° emission (open symbols) against the surface tension γ of the neat ILs at 298 K. Note that for 1 %_mol_ in [C_4_C_1_Im][Tf_2_N] and [C_8_C_1_Im][PF_6_] the signals for 0° (full) and 80° (open) fall on top of each other. Adapted from Ref.^[60]^ under CC BY license.

Pt 4f and C 1s XP spectra of 1 %_mol_ solutions of **5** in [C_2_C_1_Im][PF_6_] (black), [C_4_C_1_Im][PF_6_] (green), [C_8_C_1_Im][PF_6_] (blue) at 0° (left panel) and 80° (right panel) are depicted in Figure 14a. For comparison, the Pt 4f spectra of a 1 %_mol_ solution of **5** in [C_4_C_1_Im][Tf_2_N] (orange) are also shown. In the series of the [PF_6_]^−^‐based IL solutions, the Pt 4f signal of **5** showed a strong gradual decrease at 0° and 80° upon increasing the C_n_ chain length. This effect was also observed for all other complex‐specific signals (not shown), while the IL‐specific C_alkyl_ signals reflected an inverse trend, which is most evident in the 80° spectra: the [C_2_C_1_Im][PF_6_] solution showed only a minor C_alkyl_ intensity, whereas in [C_4_C_1_Im][PF_6_] values close to nominal were found and the [C_8_C_1_Im][PF_6_] solution showed a much higher intensity than expected from the nominal composition.^[60]^ Overall, these findings displayed a decreasing degree of surface enrichment of **5** in ILs with increasing C_n_ chain length on the IL owing to its higher surface affinity, facilitating competition of the IL for presence at the surface. The Pt 4f intensity detected from the [C_8_C_1_Im][PF_6_] solution was in line with the nominal composition of the solution, demonstrating that the surface affinity of **5** could even be suppressed to yield a homogeneous surface distribution by adequate choice of IL solvent.^[60]^

The comparison of the Pt 4f intensities detected for the [C_4_C_1_Im][PF_6_] (green) and [C_4_C_1_Im][Tf_2_N] (orange) solutions in Figure 14a immediately disclosed a much lower signal for the [Tf_2_N]^−^ solution and, consequently, a strong influence of the anion on the surface concentration of the solute.^[60]^ The intensities found for the [C_4_C_1_Im][Tf_2_N] solution quantitatively conformed with homogeneous distribution of **5** rather than surface enrichment, similar to [C_8_C_1_Im][PF_6_]. This finding was also accompanied by a slight increase of the C_alkyl_ signal at 80° for [C_4_C_1_Im][Tf_2_N] (not shown), which is indicative of a larger presence of the alkyl chains at the outer surface when compared to the solution of [C_4_C_1_Im][PF_6_], where this signal showed a slight decrease at 80°.^[60]^ Also, for equimolar solutions of only the ligand [C_3_CNPFC_4_Im][PF_6_] in [C_4_C_1_Im]Cl and [C_4_C_1_Im][PF_6_], the effect of different anions on the surface composition was successfully confirmed.^[60]^ Even though the visible difference in the XP spectra was much less obvious than found for the solutions of **5** in [C_4_C_1_Im][Tf_2_N] and [C_4_C_1_Im][PF_6_] due to the stronger surface affinity of **5** compared to the uncoordinated ligand (see above), the ligand showed a higher enrichment in [C_4_C_1_Im]Cl than in [C_4_C_1_Im][PF_6_].^[60]^

Overall, for the 1 %_mol_ solutions, the local concentration of the catalyst at the surface was strongly influenced by the length of the C_n_ chain in the [C_n_C_1_Im]^+^ cations and the nature of the anion.^[60]^ These structural features translate into different interfacial behavior, which is related to different surface tension values of the neat ILs.^[117]^ The differences in surface tension of the employed ILs are visualized in Figure 14b, depicting the temperature‐dependent surface tension curves of the neat ILs measured under ultraclean high vacuum conditions.[^60^, ^67^, ^112^] The surface tension decreases upon increasing the C_n_ chain length of the [C_n_C_1_Im][PF_6_] ILs, and regarding the variation of anions, the surface tension decreases in the order Cl^−^>[PF_6_]^−^>[Tf_2_N]^−^ for the [C_4_C_1_Im]^+^ ILs. The Pt 4f intensity detected for solutions with different ILs and concentrations was thus plotted against the surface tension values γ of the neat ILs at 298 K, as shown in Figure 14c. The higher surface enrichment of **5** (evident from higher Pt 4f intensity at both 0° and 80°) with decreasing C_n_ chain length discussed above for the 1 %_mol_ solutions of the [PF_6_]^−^ ILs (full and open black squares) excellently goes along with an increase in surface tension when decreasing the C_n_ chain length. This was assigned to the most effective lowering in surface free energy upon accumulation of the surface‐active complex **5** at the IL/vacuum interface when the surface tension of the IL is high.^[60]^ The surface tension values of [C_8_C_1_Im][PF_6_] and [C_4_C_1_Im][Tf_2_N] were even low enough to facilitate homogeneous distribution, as discussed above.^[60]^ The effects observed for the ligand‐only solutions of [C_4_C_1_Im]Cl and [C_4_C_1_Im][PF_6_] support the found impact of the IL's surface tension on the magnitude of enrichment of the solute with a much higher surface tension of 49.9 mN/m for neat [C_4_C_1_Im]Cl than that of 43.4 mN/m for neat [C_4_C_1_Im][PF_6_], at 298 K.

At higher concentrations, the solutions of **5** exhibited a different behavior. In contrast to the 1 %_mol_ solutions, 10 %_mol_ of **5** in the [PF_6_]^−^ ILs resulted in a nearly constant Pt 4f intensity at 0° and 80° (full and open blue squares in Figure 14c). Since the 10 %_mol_ solution of **5** in [C_4_C_1_Im][PF_6_] the surface was found to be saturated with the complex, as discussed above,[^53^, ^57^] this finding revealed surface saturation also in [C_2_C_1_Im][PF_6_] and [C_8_C_1_Im][PF_6_].^[60]^ Especially for the solution of [C_8_C_1_Im][PF_6_], where no surface enrichment of **5** was found at 1 %_mol_, this finding was quite surprising; apparently, a higher bulk concentration of **5** renders accumulation of the complex at the interface more favorable, even to an extent where the surface is fully saturated.^[60]^ A different behavior was found for [C_4_C_1_Im][Tf_2_N], where at 10 %_mol_ no enrichment was observed, but at 20 %_mol_ (full and open violet squares).^[60]^ It was concluded that at 10 %_mol_, the particularly low surface tension of [C_4_C_1_Im][Tf_2_N] prevented enrichment of **5**, and only increasing the concentration to 20 %_mol_ resulted in a situation where surface enrichment is more favorable than homogeneous distribution.^[60]^

## Conclusions

The present review summarized recent developments on deliberately tailoring the interfacial behavior (along with chemical aspects) of organometallic complexes in IL solutions, which lays the basis for interface‐enhanced catalysis. While the initial ligand systems investigated provided a homogeneous distribution of the catalysts or depletion from the interface, rational modifications of the ligands with surface‐active groups have shown to drastically increase the local catalyst concentration at the interface. With this as the basis, the interfacial catalyst concentration was further fine‐tuned by means of the bulk concentration, the temperature, and the design of the solvent, in specific, by tailoring its surface tension. According to the herein presented studies, a recipe for maximum surface enrichment of a catalyst would include

- attaching surface‐active groups to the ligand system acting like buoys to render surface activity, e.g. fluorinated alkyl chains or long, non‐fluorinated alkyl chains
- attaching more than one buoy to achieve more pronounced surface enrichment in a (surface) chelate‐like behavior
- using a low bulk concentration
- applying a low temperature
- choosing a solvent with high surface tension (such that the ligand‐induced decrease in surface tension of the solution is large)

First experiments on the concept of interface‐enhanced catalysts by deliberate surface enrichment of organometallic catalysts turned out to be promising since using a surface‐active catalyst has provided an activity twice as large as for a homogeneously distributed analog. Future perspectives will therefore be focused on elaborating this concept with more relevant complexes, which remain homogeneous catalysts under catalytic conditions, for more relevant conversions and higher surface areas, such as in SILP catalysis. Also, the nature of the gas atmosphere on surface enrichment phenomena is expected to have a significant influence.

## Conflict of Interests

The authors declare no conflict of interest.

1

## Biographical Information

*After completing a 3‐year apprenticeship in the chemical pigment industry, Daniel Hemmeter studied Chemistry at the Friedrich‐Alexander‐University Erlangen‐Nürnberg (FAU) and graduated in 2021. His studies were supported by the Deutschlandstipendium program and the German Academic Scholarship Foundation. Staying at FAU, he received his PhD in Chemistry as a Kekulé fellow of the Association of the Chemical Industry (VCI) in 2024, supervised by Hans‐Peter Steinrück*.

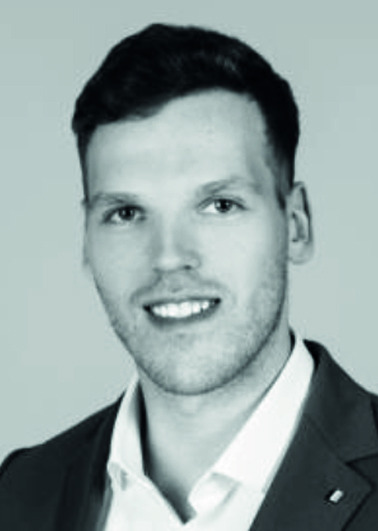

## Biographical Information

*Marco Haumann obtained his PhD in chemistry at TU Berlin in 2001. After a postdoc at Sasol Technology he became senior scientist at the Friedrich‐Alexander‐Universität Erlangen‐Nürnberg in Erlangen. His research interests are focused on the development of hybrid material for catalysis and separation, including* e.g. *supported ionic liquids (SILP and SCILL catalysis) and supported liquid alloys (SCALMS). In 2011 he was awarded the Arnold Eucken Award of the German Society of Engineers (VDI‐GVC) for his contributions to SILP technology and he is the co‐editor of the first book on Supported Ionic Liquids (Wiley‐VCH 2014). www.crt.tf.fau.eu*

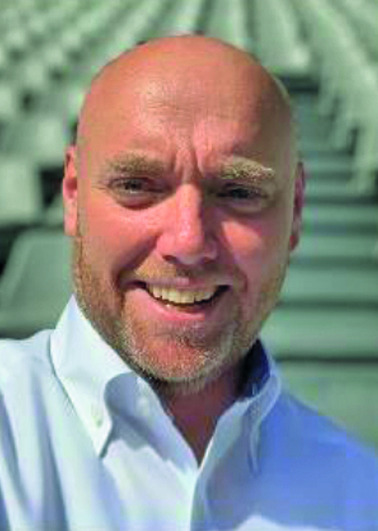

## Biographical Information

*Federico Williams is an Associate Professor at the Universidad de Buenos Aires and a Principal Researcher at the Consejo Nacional de Investigaciones Científicas y Técnicas in Argentina. He completed his PhD at the University of Cambridge in 2001, where he was an Oppenheimer Research Fellow (2000–2003) and a Trapnell Fellow at King's College (2001–2005). He also received a Leverhulme Trust Early Career Fellowship (2003–2005) during his time in Cambridge. From 2018 to 2019, he was a Guest Professor at Friedrich‐Alexander‐Universität Erlangen‐Nürnberg as a Mercator Fellow. His research focuses on surface phenomena in molecular systems with applications in energy generation and storage. http://superficies.qi.fcen.uba.ar*

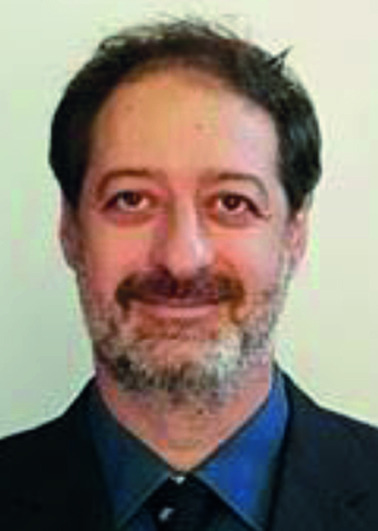

## Biographical Information

*Thomas M. Koller received his PhD in chemical engineering in 2016 and his Habilitation in thermodynamics in 2024 at the Friedrich‐Alexander‐Universität Erlangen‐Nürnberg (FAU). Since 2017, he has been an academic staff member at the Institute of Advanced Optical Technologies – Thermophysical Properties (AOT‐TP) of FAU. He is a member of the International Association for Transport Properties and in the editorial board of the International Journal of Thermophysics. The research of his group focuses on the characterization of multiphase systems in chemical end energy engineering by investigating their thermophysical properties. www.aot‐tp.tf.fau.de/person/thomas‐koller/*

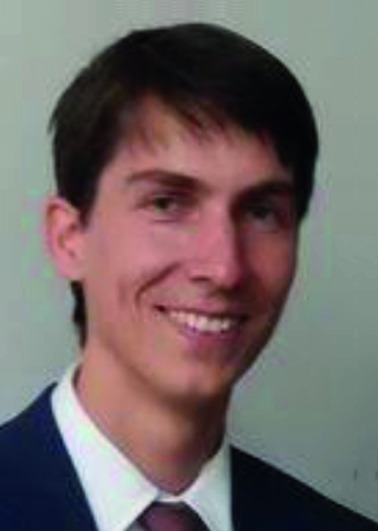

## Biographical Information

*Karsten Meyer studied chemistry at the Ruhr University Bochum and earned his Ph.D. in 1998 under Karl Wieghardt at the Max Planck Institute in Mülheim/Ruhr. He conducted postdoctoral research with Christopher Cummins at MIT. In 2001, he was appointed Assistant Professor at the University of California, San Diego, and became an Alfred P. Sloan Fellow in 2004. In 2006, he joined FAU Erlangen‐Nürnberg as Chair of Inorganic and General Chemistry. His research focuses on synthetic d‐ and f‐block metal complexes within custom‐tailored ligand architectures, developing platforms for charge and light‐driven catalysis, advancing fundamental reactivity, and exploring new small‐molecule activation strategies. www.inorgchem2.nat.fau.de*

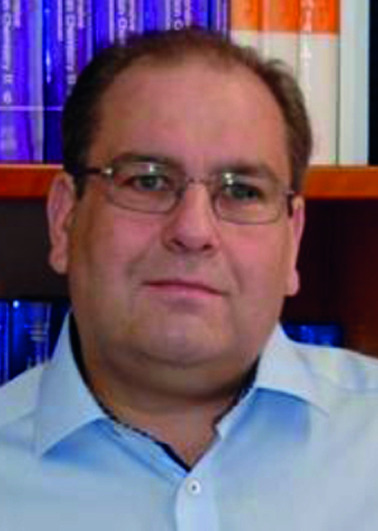

## Biographical Information

*Peter Wasserscheid heads the Institute of Chemical Reaction Engineering at Friedrich‐Alexander‐Universität Erlangen‐Nürnberg and is director at Forschungszentrum Jülich. He studied chemistry at RWTH Aachen and finished his PhD in 1998. After an industrial postdoc with BP Chemicals he completed his Habilitation at RWTH Aachen and joined FAU in 2003. The director position at Forschungszentrum Jülich added in 2014 to his duties. Peter's research focusses on the science and technology of ionic liquids and chemical hydrogen storage systems. In 2006, he received the Leibniz Award of the German Science Foundation and later two Advanced Grants of the European Research Council in 2010 and 2018. www.crt.tf.fau.eu, www.hi‐ern.de/en, www.hch2.de/en/*

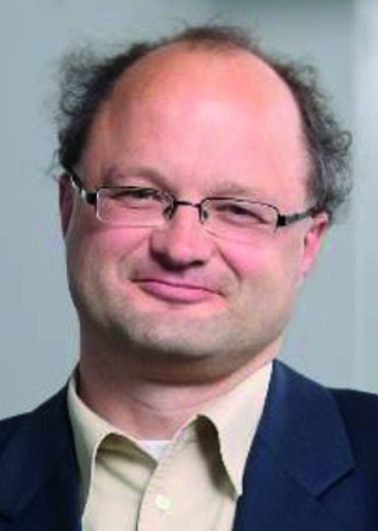

## Biographical Information

*F. Maier (1969) studied physics at the universities of Würzburg and Grenoble. At the FAU, he completed his PhD on electronic properties of diamond interfaces with L. Ley and became postdoc at the Chair of Physical Chemistry II within H.‐P. Steinrück's group. He obtained a permanent position 2003, where he is now Academic Director. Since 2005, he has been leading the Ionic Liquid Surface Science (ILSS) group. His work involves developing innovative methods and advanced instrumentation for investigating surfaces and interfaces of ionic liquid systems under ultrahigh vacuum conditions. He has authored more than 80 publications in this field. www.chemistry.nat.fau.eu/maier‐group/*

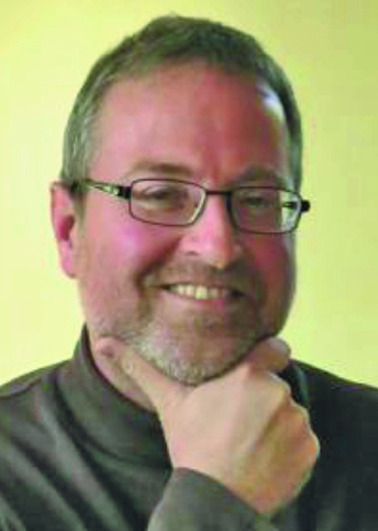

## Biographical Information

*Hans‐Peter Steinrück did his PhD in physics at TU Graz 1985, was postdoc at Stanford University, received his Habilitation at TU München, and became Professor of Physics at Würzburg University in 1993. Since 1998, he holds a chair of Physical Chemistry at the Friedrich‐Alexander‐Universität Erlangen‐Nürnberg. He is member of the European Academy of Sciences, German Academy of Sciences Leopoldina, Austrian Academy of Sciences, and Academia Europaea. In 2016, he received an ERC Advanced Grant. His research focusses on surface and interface science, from ionic liquids, porphyrins, liquid metals and liquid organic hydrogen carriers to chemically modified graphene. www.chemistry.nat.fau.eu/steinrueck‐group/*

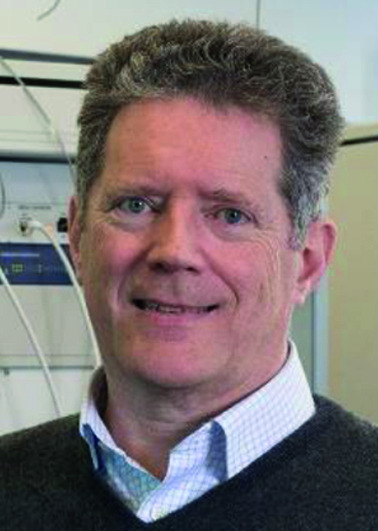

## Data Availability

Data sharing is not applicable to this article as no new data were created or analyzed in this study.
